# Mucin 2 silencing promotes colon cancer metastasis through interleukin-6 signaling

**DOI:** 10.1038/s41598-017-04952-7

**Published:** 2017-07-19

**Authors:** Hui-Ping Hsu, Ming-Derg Lai, Jenq-Chang Lee, Meng-Chi Yen, Tzu-Yang Weng, Wei-Ching Chen, Jung-Hua Fang, Yi-Ling Chen

**Affiliations:** 1 Department of Surgery, National Cheng Kung University Hospital, College of Medicine, National Cheng Kung University, Tainan, Taiwan; 2 Department of Biochemistry and Molecular Biology, College of Medicine, National Cheng Kung University, Tainan, Taiwan; 3 Institute of Basic Medical Sciences, College of Medicine, National Cheng Kung University, Tainan, Taiwan; 4 Department of Emergency Medicine, Kaohsiung Medical University Hospital, Kaohsiung Medical University, Kaohsiung, Taiwan; 5 Laboratory Animal Center, College of Medicine, National Cheng Kung University, Tainan, Taiwan; 6 Department of Senior Citizen Service Management, Chia Nan University of Pharmacy and Science, Tainan, Taiwan; 7 Senior Citizen Development Center, Chia Nan University of Pharmacy and Science, Tainan, Taiwan

## Abstract

Downregulation of Mucin 2 (MUC2) expression is associated with early carcinogenesis events in colon cancer. MUC2 plays a role in the progression of colon cancer, and reduced MUC2 protein expression correlates with increased interleukin-6 (IL-6) expression. However, the interaction between MUC2 and IL-6 in colorectal cancer metastasis remains unclear. We systematically analyzed MUC2 and IL-6 expression and determined the survival of cancer patients with high or low MUC2 and IL-6 expression using the Oncomine and PrognoScan databases, respectively. This analysis identified downregulation of *MUC2* and overexpression of *IL-6* in colon cancer but not in normal colon tissue, and this expression pattern was correlated with poor survival of colon cancer patients. We examined the effects of MUC2 on colon cancer metastasis and used vector-mediated application of short hairpin RNA (shRNA) to suppress MUC2 expression. MUC2 suppressed the migration of colon cancer cells *in vitro* and dramatically diminished liver metastases *in vivo*. Treatment with IL-6 increased signal transducer and activator of transcription 3 (STAT3) phosphorylation, promoted checkpoint kinase 2 (Chk2) activation, attenuated cAMP response element-binding protein (CREB) phosphorylation, and suppressed E-cadherin protein expression in MUC2-silenced HT-29 cancer cells. Most importantly, MUC2 is a potential prognostic indicator for colon cancer.

## Introduction

The secretory protein mucin 2 (MUC2) is the primary component of the protective mucus layer of the colon and plays a role in the progression of colorectal cancer (CRC)^1^. MUC2 expression is attenuated in nonmucinous colon adenocarcinomas (ACs) but preserved in mucinous colon carcinomas. Previous studies have reported that the loss of MUC2 expression is a poor prognostic factor in stage II and III colorectal carcinoma^2^. Loss of MUC2 expression is observed in CRC and is associated with progression and metastasis^3–5^. Moreover, MUC2 expression is correlated with better survival and a decreased incidence of liver and nodal metastasis in colon AC^2, 6^. Furthermore, MUC2 expression tends to predict a good prognosis in well-to-moderately differentiated AC but not in poorly differentiated AC^7, 8^. Altered MUC2 expression may contribute to changes in the invasive and metastatic capabilities of the cancer^9, 10^. Therefore, MUC2 may play an important role in colon carcinoma metastasis and predict cancer recurrence.

Various studies have reported that elevated IL-6 levels are associated with advanced tumor stages, increased tumor size, metastasis, and decreased survival of CRC patients^11–14^. Low preoperative levels of IL-6 are significantly associated with a longer disease-free survival in stage I-III colon cancer patients^15^. In addition, high serum IL-6 is a risk factor for CRC recurrence, including in stage II patients^15^. IL-6 is an important mediator of the tumor-promoting effects of inflammation^16^. In previous studies, tumor-infiltrating macrophages were shown to express a high level of IL-6^17, 18^. Understanding IL-6-mediated signaling may facilitate the design of better cancer therapeutics.

MUC2 suppression enhanced IL-6 secretion and tumor growth in a colon cancer animal model^19^. To identify the best *in vitro* models for studying mucin 2 expression, western blotting was used to measure the expression of MUC2 in the cell lines HT-29, LS174T, SW620, Colo205, and LoVo. A previous study reported that LS174T is a goblet-cell-like CRC cell line^20^. The human colon AC cell line HT-29 is commonly used as a model of epithelial cell differentiation in CRC, and the elucidation of a regulatory mechanism in HT-29 cells might have important clinical implications for the treatment of colon cancer^21^. Therefore, we used HT-29 as a model cell line to study MUC2 signaling in colon AC. We knocked down the expression of MUC2 using shRNA. The objective of this study was to examine the signaling pathways mediated by the interaction between MUC2 and IL-6 during colon cancer metastasis.

## Results

### Analysis of *MUC2* and *IL-6* gene expression

We previously demonstrated that MUC2 suppression enhances IL-6 secretion in the colon cancer cell line CT26^19^. To further investigate the interaction between MUC2 and IL-6 in colon cancer, we compared the expression of *MUC2* and *IL-6* in normal colon and colon cancer tissues using the Oncomine database. Downregulation of *MUC2* and overexpression of *IL-6* were found in colon cancer (Supplementary Figures S1–S2). *MUC2* gene expression was significantly decreased in several types of colon cancer, including AC, CRC, rectal AC, and cecum AC (Supplementary Figure S1). *IL-6* gene expression was significantly increased in colon adenoma, CRC, colon carcinoma, rectal AC, colon mucinous carcinoma, colon AC, cecum AC, and rectosigmoid AC (Supplementary Figure S2). Thus, our Oncomine analysis of colon cancer identified downregulation of *MUC2* and overexpression of *IL-6* in colon cancer but not in normal colon tissue.

### Association of *MUC2* and *IL-6* expression with survival in colon cancer patients

**Figure 1:**
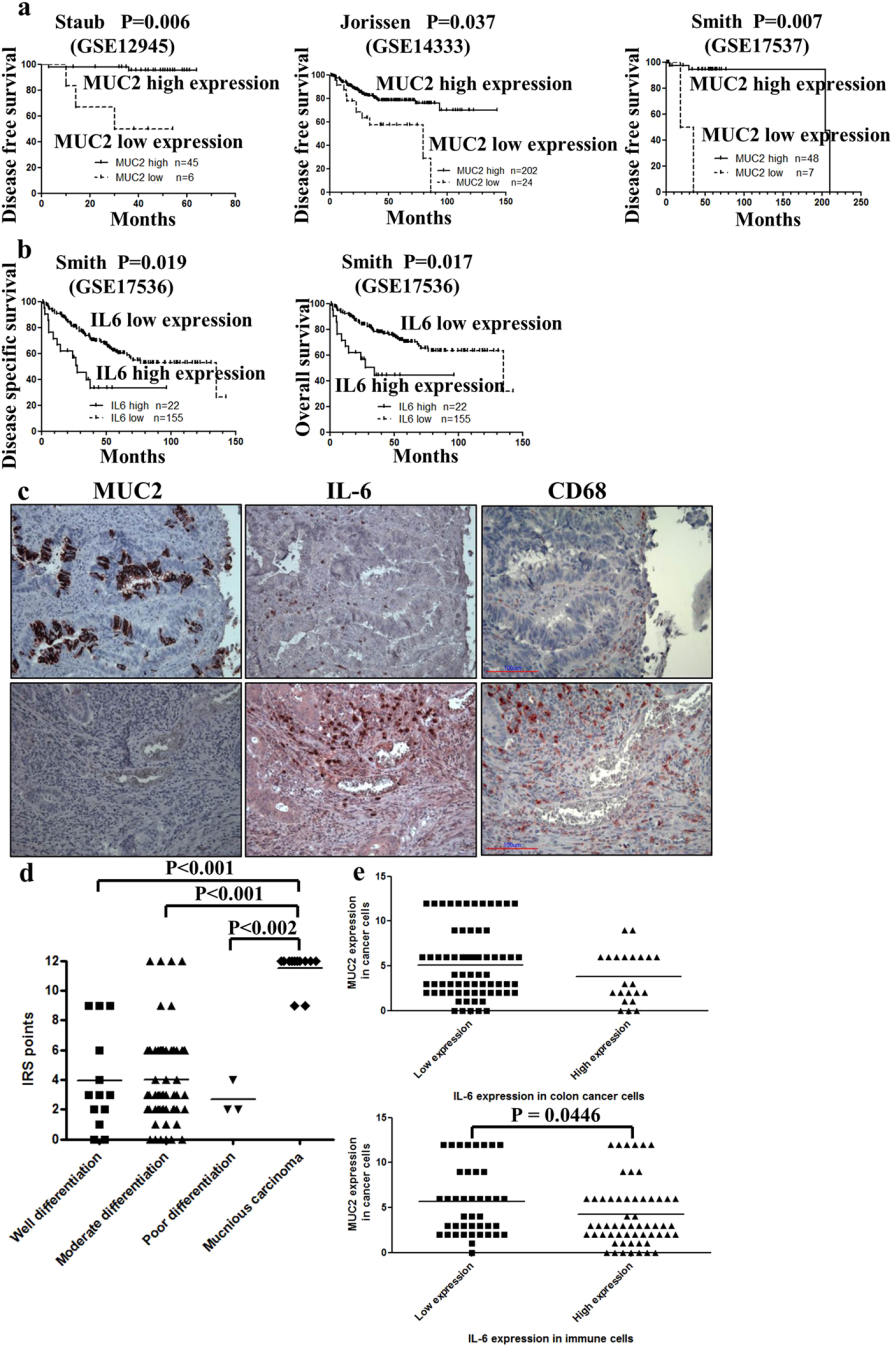
Kaplan–Meier survival curves relative to *mucin 2* (*MUC2)* and interleukin 6 (*IL-6)* gene expression in patients with colon cancer. (**a**) Disease-free survival (DFS) is analyzed with respect to *MUC2* expression levels. (**b**) Disease-specific survival (DSS) and overall survival (OS) are analyzed with respect to *IL-6* expression levels. The total number of patients in the low and high expression groups and *P-*values are shown. The high and low *MUC2* expression groups were determined using the best cutoff value according to the PrognoScan database description. The x-axis represents time, and the y-axis represents DFS (**a**), DSS (**b**), and OS (**b**). (**c**) Immunohistochemical staining to determine the distribution of MUC2-positive cells (red), CD68-positive cells (red) and IL-6-positive cells (red) in paraffin-embedded specimens from AJCC stage IIA colon cancer patients (magnification, ×200). AJCC, American Joint Committee on Cancer. A stage IIA colon cancer specimen with high MUC2 expression and low IL-6 and CD68 expression (magnification, ×200) (upper). A stage IIA colon cancer specimen with low MUC2 expression and high IL-6 and CD68 expression (magnification, ×200) (lower). (**d**) Expression of MUC2 and differentiation type in stage IIA colon cancer as assessed by immunohistochemical (IHC) staining and scaling according to the immunoreactive score (IRS) of Remmele and Stegner, as follows: 0–1, negative expression; 2–3, weak expression; 4–8, mild expression; 9–12, strong expression. (**e**) Correlation between MUC2 and IL-6 expression in colon cancer cells of patients with stage IIA colon cancer (upper). Patients with a lower level of MUC2 expression specifically had higher immune cell infiltration (lower).

The relationship between MUC2 and IL-6 gene expression and colon cancer patient prognosis was examined using the PrognoScan database. High *MUC2* gene expression correlated with better disease-free survival (DFS) in Kaplan–Meier survival curves (Fig. 1a). High *IL-6* gene expression correlated with worse disease-specific survival (DSS) and overall survival (OS) (Fig. 1b). Thus, our bioinformatic analyses using the Oncomine and PrognoScan databases indicate that MUC2 and IL-6 expression are involved in cancer progression. These results demonstrate that downregulation of the *MUC2* gene and overexpression of the *IL-6* gene are predictors of poor prognosis in colon cancer patients.

### Immunohistochemical analysis of MUC2, IL-6, and CD68 in human colon cancer

We used immunohistochemistry to detect the protein expression of MUC2, IL-6, and CD68 in serial sections of colon cancer tissues. CD68 is expressed on macrophages^22^. We determined the MUC2 and IL-6 expression levels in 102 patients with stage IIA colon cancer and their correlation with clinicopathologic characteristics (Supplementary Table 1). MUC2 and IL-6 were localized in the cytoplasm of the cancer cells (Fig. 1c). Strong MUC2 expression and low IL-6 and CD68 expression were detected in the same specimen from a patient with stage IIA cancer (Fig. 1c, upper left and upper right). Loss of MUC2 and strong expression of IL-6 and CD68 were also detected in serial sections of one specimen from another patient with stage IIA cancer (Fig. 1c, lower left and lower right). Most of the IL-6-positive cells were macrophages.

Patients with mucinous carcinoma of the colon had a higher level of MUC2 expression than did those with well, moderately, and poorly differentiated AC (Fig. 1d). Patients with a lower level of MUC2 expression had a trend of higher IL-6 expression in colon cancer cells and specifically higher infiltration by immune cells **(**P = 0.0446**)** (Fig. 1e). A correlation between the IL-6 secretion of CD68 macrophages and a higher level of IL-6 in cancer cells was observed. Most importantly, inverse expression of the MUC2 and IL-6 proteins was observed in patients with stage II colon cancer.

### Suppressing MUC2 expression did not influence cell proliferation in HT-29 and LS174T human colon cancer cells

**Figure 2:**
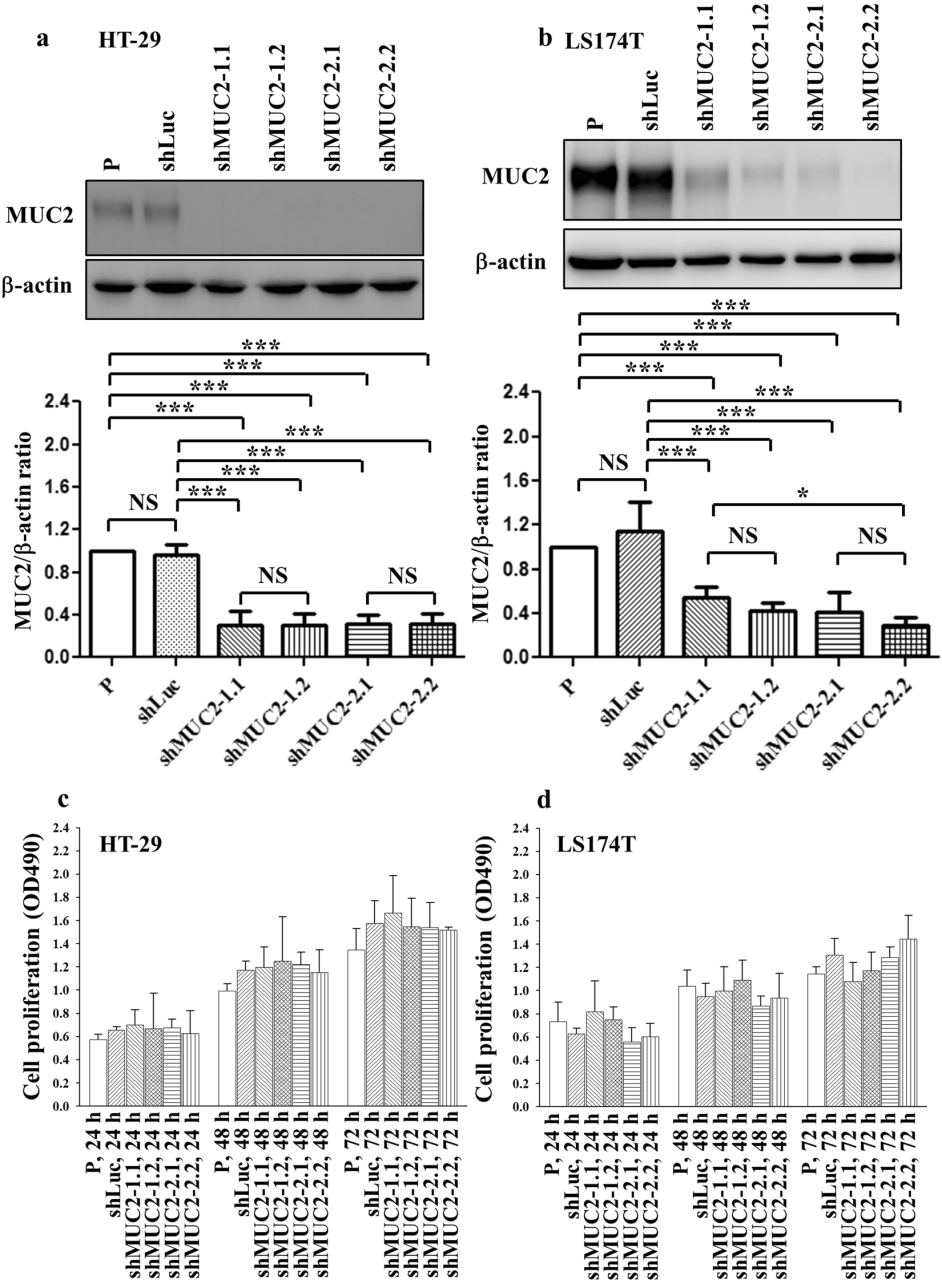
MUC2 silencing in HT-29 and LS174T cells did not influence cell proliferation. (**a** and **b**) MUC2 protein expression was determined in human HT-29 and LS174T cells and MUC2 shRNA stable transfectants. The results of the western blot analysis of protein expression were obtained from three independent experiments. The bars represent the mean ± the SD. P, parental cells; shLuc, luciferase control; shMUC2-1.1, shMUC2-1.2, shMUC2-2.1 and shMUC2-2.2, MUC2-specific shRNAs 1 and 2, respectively. (**c** and **d**) The proliferation of HT-29 and LS174T cells and MUC2 shRNA stable transfectants was determined at 24, 48, and 72 h. NS, not significant; *P < 0.01; **P < 0.001; ***P < 0.0001.

We first analyzed the MUC2 protein expression in five human colon cancer cell lines (HT-29, LS174T, SW620, Colo205, and LoVo). Of the five cell lines examined, LS174T and HT-29 cells constitutively express MUC2 (Supplementary Figure S3a and b). MUC2 protein expression was significantly higher in LS174T cells than in HT-29 cells and all other cancer cell lines tested (Supplementary Figure S3b). We knocked down the expression of MUC2 using two different shRNAs. We established four stable cell lines, with two clones for each of the two RNAi constructs: shMUC2-1.1, shMUC2-1.2, shMUC2-2.1, and shMUC2-2.2. Successful suppression of the MUC2 protein in HT-29 (Fig. 2a; Supplementary Figure S4a) and LS174T (Fig. 2b; Supplementary Figure S4b) cancer cells was demonstrated by western blotting. The growth rates of the MUC2-silenced HT-29 (Fig. 2c) and LS174T cells (Fig. 2d) were not altered by MUC2 shRNA. Therefore, suppression of MUC2 did not affect cell growth in the HT-29 and LS174T human cancer cell lines.

### MUC2-silenced HT-29 cells exhibit increased IL-6 secretion upon treatment with macrophage supernatant

Previous studies have shown that suppression of MUC2 expression increases IL-6 secretion and that the presence of IL-6 in macrophage supernatants (MSs) induces IL-6 secretion by HT-29 cells^19, 21^. No IL-6 was detected in supernatants from the HT-29 parental cells and HT-29-derived cell clones. To determine whether MUC2 silencing increases IL-6 secretion in HT-29 colon cancer cells, we incubated HT-29-derived cell clones with MSs for 48 h and determined the IL-6 concentration in the resulting supernatants by ELISA. CD14^+^ macrophages were isolated from peripheral blood mononuclear cells (PBMCs), and the cell purity was >90% (Supplementary Figure S5a). The concentration of IL-6 in conditioned medium from MS-treated shMUC2-1.2 and shMUC2-2.2 cells was significantly higher than that from MS-treated parental cells or MS-treated shLuc cells (Supplementary Figure S5b). To verify the role of IL-6, the IL-6 derived from the MS was neutralized by treating the MS with 5 µg/ml anti-IL-6 antibody for 30 min before adding the MS to the HT-29-derived cells. These results indicated that the IL-6 present in the MS acts as an inducing factor for the expression of IL-6 in colon cancer cells. The expression of glycoprotein 130 (gp130), an important receptor in IL-6 signal transduction, was determined using flow cytometry (Supplementary Figure S5c). The silencing of MUC2 in HT-29 cells (shMUC2-1.2 and shMUC2-2.2 cells) increased gp130 expression (Supplementary Figure S5d). Thus, MUC2 expression in colon cancer may play an important role in the IL6-dependent signaling pathway.

### Suppression of MUC2 increased the migration of HT-29 and LS174T cells

**Figure 3:**
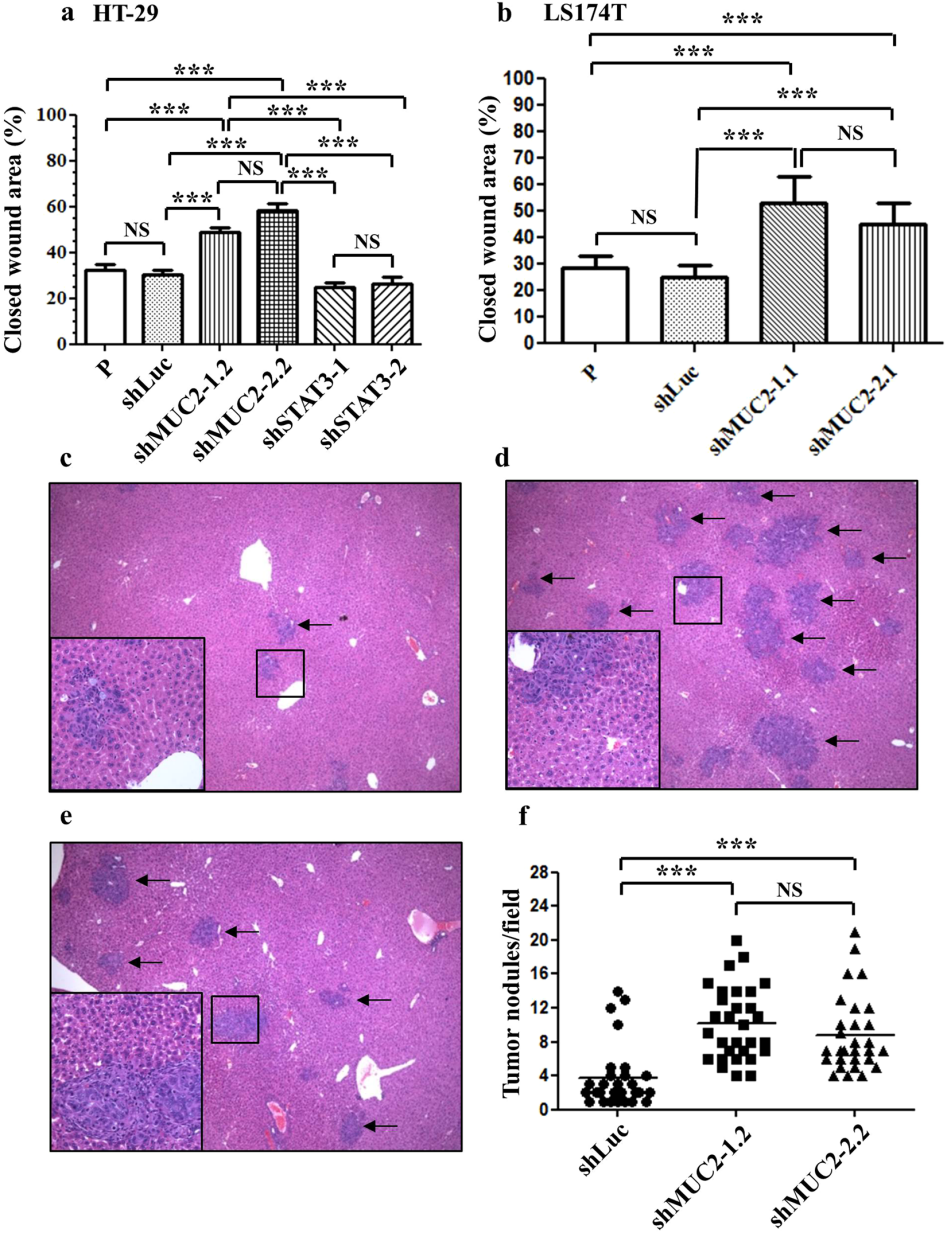
MUC2 silencing increased cell migration/metastasis and STAT3 silencing inhibited cell migration in HT-29 cell clones. (**a**) MUC2-silenced and STAT3-silenced HT-29 cells were examined using the wound-healing assay. (**b**) MUC2-silenced LS174T cells were examined using the wound-healing assay. The data represent quantitative results of the *in vitro* wound-healing assay at 48 h and are presented as the mean ± the SD of three independent experiments. (**c**–**e**) Hematoxylin and eosin staining of liver sections (magnification, ×40); macrometastases are indicated by arrows. Liver metastasis was observed in mice injected with colon cancer cells transfected with shLuc (**c**), shMUC2-1.2 (**d**) and shMUC2-2.2 (**e**). The boxed areas in (**c**), (**d**) and (**e**) are shown at higher magnification (×100). (**f**) The number of tumor nodules per field of liver in NOD/SCID mice was determined on postinjection day 14. The data are expressed as the mean ± the SD of two independent experiments. P, parental cells; shLuc, luciferase control; shMUC2-1.2 and shMUC2-2.2, MUC2-specific shRNAs 1 and 2, respectively; NS, not significant; *P < 0.01; **P < 0.001; ***P < 0.0001.

A wound-healing assay was used to examine the migratory ability of HT-29 and LS174T cells *in vitro*. Suppression of MUC2 increased cell migration at 48 h in HT-29 cells (Fig. 3a; Supplementary Figure S6a) and LS174T cells (Fig. 3b; Supplementary Figure S6b). The downregulation of MUC2 in HT-29 and LS174T cells by two different shRNAs increased migration.

Constitutive signal transducer and activator of transcription 3 (STAT3) activation in CRC cells is associated with invasion^23^. Therefore, we used shRNA to suppress STAT3 expression of in the HT-29 colon cancer cell line. Downregulation of STAT3 protein expression was verified by western blotting. STAT3 expression was significantly lower in shSTAT3-1 and shSTAT3-2 HT-29 cells than in control cells (Supplementary Figure S7a and b). shSTAT3-1- and shSTAT3-2-transfected HT-29 cells exhibited a relatively low migration potential (Fig. 3a; Supplementary Figure S6a).

### Suppression of MUC2 promoted liver metastasis of HT-29 cells

A mouse model of liver metastasis was used to examine the function of MUC2 in colon cancer. Liver metastases were established following intrasplenic injection of HT-29 cells in immunocompromised nonobese diabetic/severe combined immunodeficient (NOD/SCID) mice (Fig. 3c–f). Hematoxylin and eosin staining was used to examine the liver metastases (Fig. 3c–e). The number of liver metastases was significantly lower in mice injected with shLuc cells than in those injected with shMUC2-1.2 and shMUC2-2.2 cells (Fig. 3f). Thus, MUC2 silencing promotes metastasis in colon cancer.

### Suppression of MUC2 promoted liver metastasis of CT26 cells

In a previous study, we established three clones of MUC2-suppressed cells: CT26 MUC2 RNAi-1, CT26 MUC2 RNAi-2, and CT26 MUC2 RNAi-3^19^. To further confirm the function of MUC2 in cancer metastasis, we examined the changes in liver metastasis induced by intrasplenic injection of colon cancer cells in immunocompetent BALB/c mice. We knocked down the expression of MUC2 using two different shRNAs. We established two stable cell clones: MUC2 RNAi-1 and MUC2 RNAi-2^19^. Successful suppression of MUC2 proteins in CT26 cancer cells was demonstrated by western blotting (Supplementary Figure S8a). The weights of the livers were higher in the CT26 MUC2 RNAi-1 and CT26 MUC2 RNAi-2 groups (Supplementary Figure S8b and c). We used the CT26 mouse model to demonstrate that the metastatic burden was higher in mice injected with the MUC2-suppressed cells. Taken together, our results indicate that suppression of MUC2 promoted liver metastasis in an animal model.

### MUC2 plays a role in IL-6-mediated activation of downstream signaling pathways

**Figure 4:**
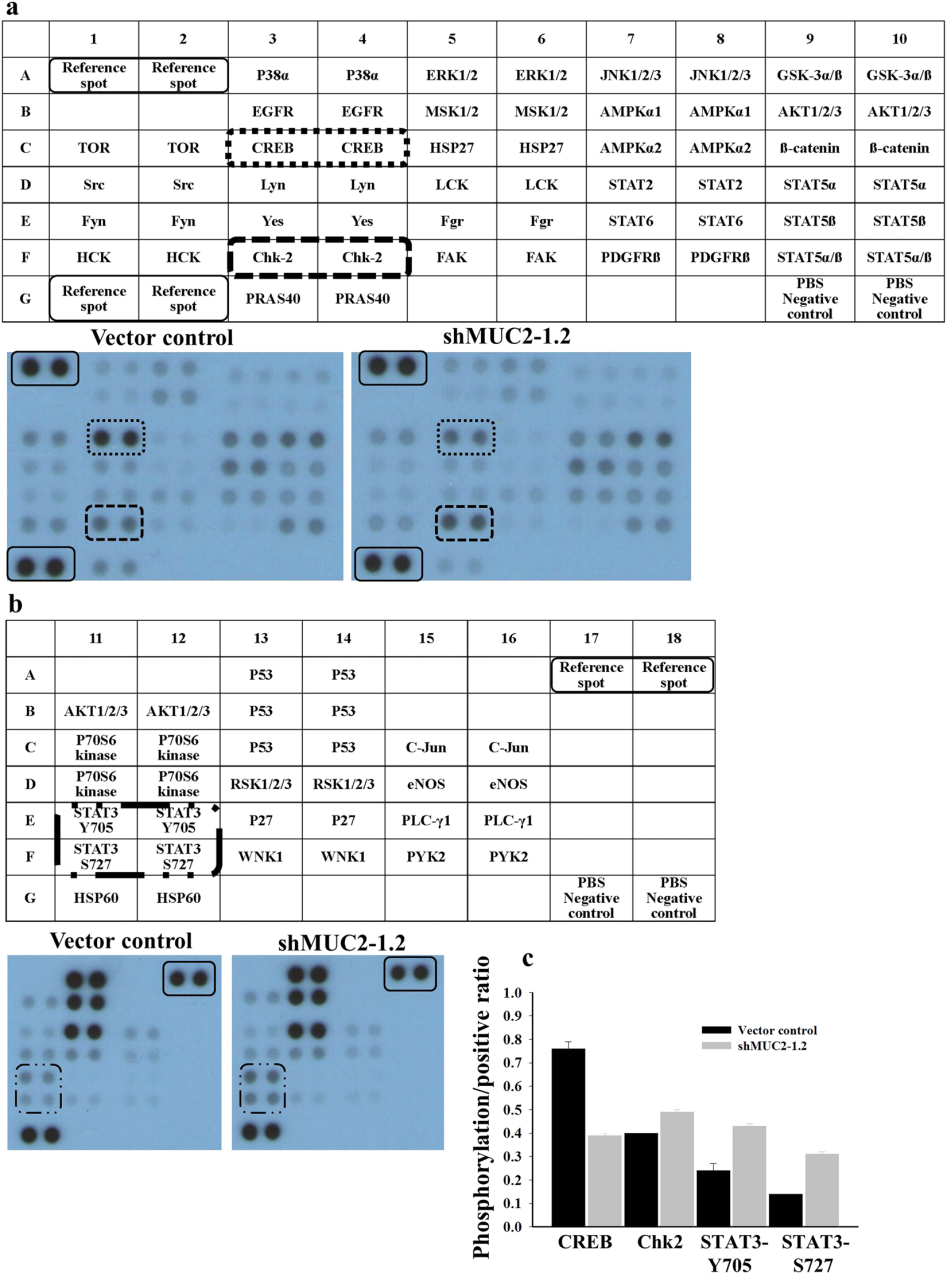
Profiling phosphor-signaling networks in HT-29-derived cells after exogenous treatment with recombinant human IL-6. To detect intracellular signaling, the activation of important signaling components was observed using human Phospho-RTK Arrays. MUC2 suppression leads to decreased CREB phosphorylation (**a**) and increased STAT3 (**b**) and Chk2 phosphorylation (**a**) in HT-29 colon cancer cells exogenously supplemented with human rIL-6. (**c**) Bars represent the mean ± the SD. shMUC2-1.2, MUC2-specific shRNA 1.

In previous studies, both autocrine and paracrine IL-6 signaling increased the invasive and metastatic ability of human cancer cells^24^. To identify potential downstream proteins in the IL-6-dependent signaling pathway, the Proteome Profiler Human Phospho-Kinase Array was used to analyze kinase signaling. HT-29 cells were treated with recombinant human IL-6 (rIL-6), and the kinase activation patterns were compared between vector control and MUC2-silenced cells. STAT3 (Tyr 705) and checkpoint kinase 2 (Chk2) (Thr 68) phosphorylation increased in MUC2-silenced HT-29 cells after rIL-6 treatment (Fig. 4a–c). The level of phosphorylated CREB (Ser133) decreased in MUC2-silenced HT-29 cells after IL-6 treatment (Fig. 4a and c). Suppression of MUC2 expression thus promotes IL-6-mediated phosphorylation of STAT3/Chk2 and suppresses CREB phosphorylation.

**Figure 5:**
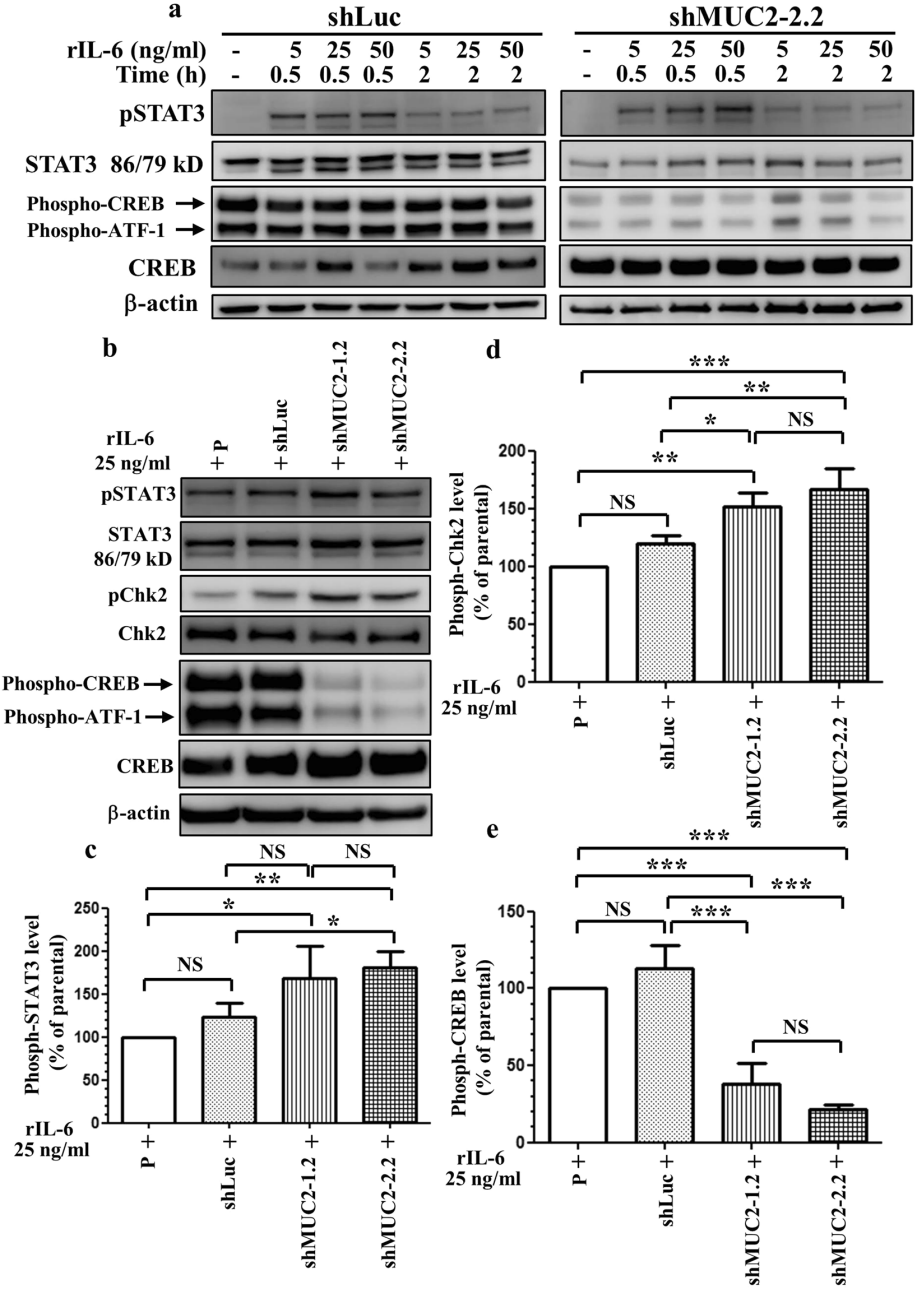
Exogenous rIL-6 induces IL-6-dependent signaling in HT-29-derived cells. (**a**) Time-course and dose-response analysis of HT-29-derived cells with or without recombinant human IL-6 treatment. (**b**–**e**) Increased STAT3/Chk2 phosphorylation and decreased CREB phosphorylation were detected in MUC2-silenced HT-29 cells after exogenous treatment with recombinant human IL-6. (**b**) Western blot analysis of STAT3, Chk2, and CREB in HT-29-derived cells. MUC2-specific shRNA increased the phosphorylation levels of STAT3 (**c**) and Chk2 (**d**) and decreased the phosphorylation levels of CREB (**e**) in HT-29 cells treated with exogenous human rIL-6. The data represent the mean ± the SD of three independent experiments. STAT3 isoform expression appears as STAT3α (86 kDa) and STAT3β (79 kDa). P, parental cells; shLuc, luciferase control; shMUC2-1.2 and shMUC2-2.2, MUC2-specific shRNAs 1 and 2, respectively; NS, not significant; **P < 0.001; ***P < 0.0001.

The signaling changes identified by the phospho-kinase array were confirmed by western blotting. We examined whether STAT3 phosphorylation in HT-29-derived cells was induced by IL-6. We found that STAT3 was activated in a concentration-dependent manner after stimulation with IL-6; the STAT3 activation was transient, reaching a maximum 30 min after stimulation (Fig. 5a; Supplementary Figure S4c). STAT3 activation peaked 30 min after IL-6 treatment in shLuc and shMUC2-2.2 cells. Specifically, STAT3 and Chk2 phosphorylation was significantly higher in IL-6-treated shMUC2-1.2 cells and shMUC2-2.2 cells than in IL-6-treated shLuc cells (Fig. 5a–d; Supplementary Figure S4c and d). CREB/ATF-1 phosphorylation was significantly lower in IL-6-treated shMUC2-1.2 cells and shMUC2-2.2 cells than in IL-6-treated shLuc cells (Fig. 5b and e; Supplementary Figure S4e). Our experiments showed that IL-6 treatment induced STAT3 and Chk2 activation and attenuated CREB/ATF-1 phosphorylation in MUC2-silenced HT-29 cancer cells.

We therefore assessed the protein expression of CREB, STAT3, and Chk2 in the LS174T and HT-29 cell lines, which are derived from the primary tumors of colorectal ACs, and in three cell lines (LoVo, SW620 and Colo205) established from metastatic sites of colorectal ACs. All five cell lines constitutively expressed Chk2 (Supplementary Figure S3c). The HT-29, LS174T, and SW620 cell lines highly expressed CREB (Supplementary Figure S3c and d). CREB expression was not detected in Colo205 cells (Supplementary Figure S3c and d). The LoVo and Colo205 cell lines highly expressed STAT3 (Supplementary Figure S3c and e). Distinct CREB and STAT3 expression patterns were observed in the colon cancer cell lines, indicating that CREB protein expression is inversely correlated with that of STAT3 in colon cancer.

### Functional loss of E-cadherin in MUC2-silenced HT-29 cells

**Figure 6:**
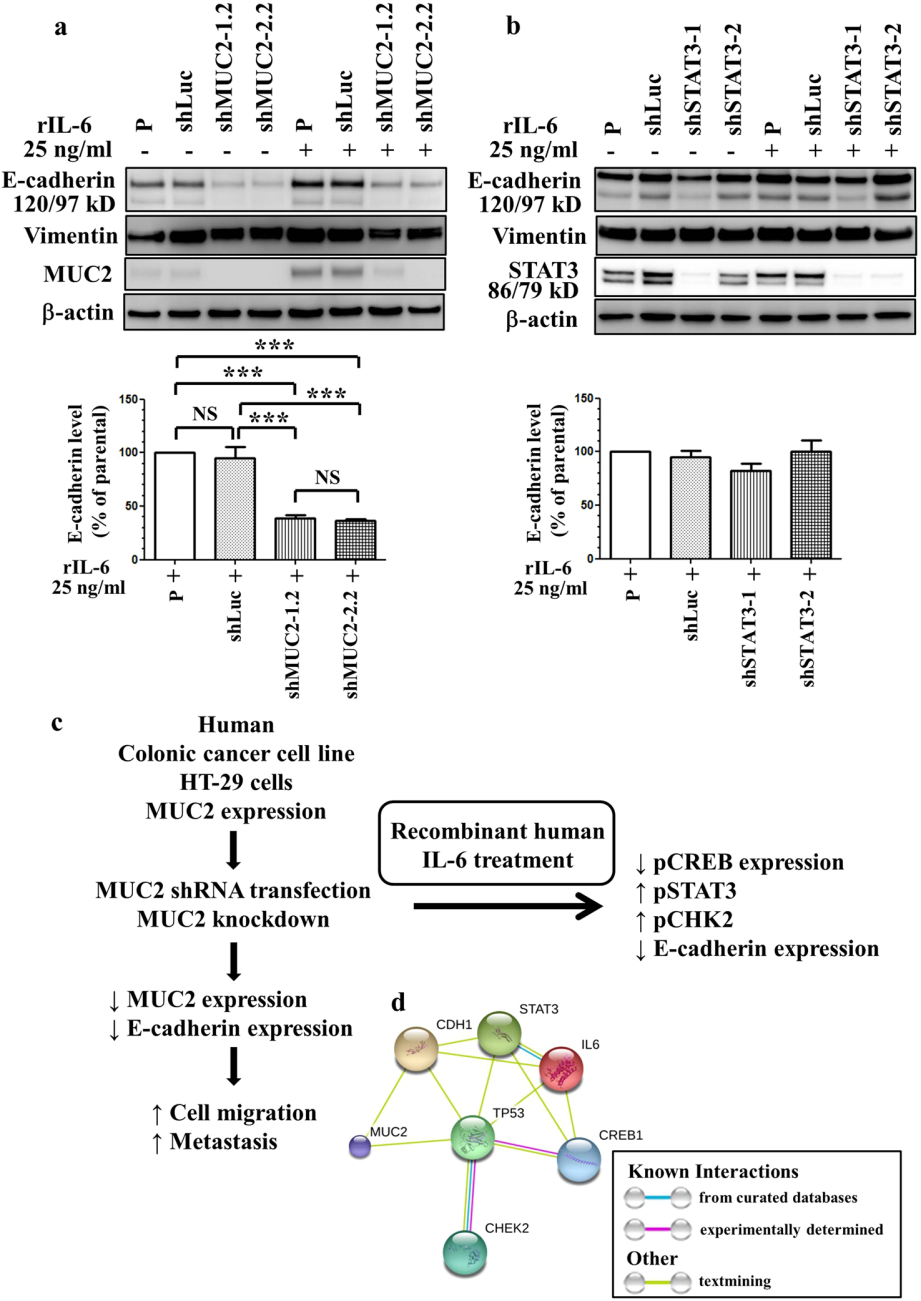
Expression of E-cadherin in HT-29-derived cells with or without recombinant human IL-6 treatment. HT-29-derived cells were treated with rIL-6 for 30 min. E-cadherin, vimentin, MUC2, and STAT3 expression levels were examined in IL-6-treated HT-29-derived cells by western blotting. (**a**) Loss of E-cadherin expression in MUC2-silenced HT-29 cells with or without recombinant human IL-6 treatment for 30 min. (**b**) Significantly reversed E-cadherin expression in STAT3-silenced HT-29 cells with or without recombinant human IL-6 treatment for 30 min. P, parental cells; shLuc, luciferase control; shMUC2-1.2 and shMUC2-2.2, MUC2-specific shRNAs 1 and 2, respectively; shSTAT3-1 and shSTAT3-2, STAT3-specific shRNAs 1 and 2, respectively; NS, not significant; **P < 0.001; ***P < 0.0001. (**c**) Proposed model depicting the effects of MUC2 and IL-6 treatment on cell migration and metastasis in HT-29 colon cancer cells. (**d**) Protein interaction network of MUC2, IL-6, STAT3, CREB1, CHEK2, CDH1, and TP53. The colored lines between the proteins indicate the various types of evidence demonstrating the interaction. The evidence for these interactions is derived from both experimental evidence (purple lines) and text-mining evidence (green lines). MUC2: mucin 2; TP53: Tumor protein p53; CREB1: cAMP responsive element-binding protein 1; CDH1: epithelial cadherin (E-cadherin); STAT3: Signal transducer and activator of transcription 3; IL-6: Interleukin 6; CHEK2: Checkpoint kinase 2.

We hypothesized that IL-6 induces invasion and metastasis of primary colon cancer through the epithelial-mesenchymal transition (EMT) process. Loss of E-cadherin in epithelial cells is a hallmark of EMT^25^. E-cadherin expression was suppressed by rIL-6 treatment and by MUC2 silencing in HT-29 cells (Fig. 6a; Supplementary Figure S9a).

STAT3 phosphorylation was induced by IL-6 and further augmented in MUC2-silenced cells. To examine whether IL-6-mediated EMT occurred through STAT3 activation, we knocked down STAT3 using shRNA. Knockdown of MUC2 or STAT3 did not significantly alter vimentin levels (Fig. 6a,b). Exploring the phosphorylation of CREB and STAT3 in relation to MUC2 might reveal associations between MUC2 and IL-6 in colon cancer. Knockdown of STAT3 in HT-29 cells significantly reversed the IL-6-mediated suppression of E-cadherin (Fig. 6b; Supplementary Figure S9b) and did not affect CREB phosphorylation (Supplementary Figure S9c and d). We observed suppressed E-cadherin expression and decreased CREB/ATF-1 phosphorylation in MUC2-silenced tumor cells after rIL-6 treatment (Supplementary Figure S9c and d). These data indicate that MUC2 silencing in cancer cells suppressed E-cadherin and pCREB expression. The effects of MUC2 and IL-6 on colonic cancer cell migration and metastasis are summarized in Fig. 6c. We wanted to exploit protein interaction data to represent significant protein interactions in colon cancer. Relevant protein–protein interaction information was retrieved from the Search Tool for the Retrieval of Interacting Genes (STRING) database website (Fig. 6d), revealing a highly interconnected network. STRING predicts protein–protein interactions derived from sources such as high-throughput experiments, co-expression data, and literature reports. Evidence for physical interactions between MUC2, TP53, CREB1, CDH1 (E-cadherin), STAT3, IL-6, and CHEK2 (Chk2) was found in the STRING database when querying MUC2/IL-6 and was based on evidence from text mining.

## Discussion

Our previous study demonstrated that the suppression of MUC2 in CT26 colon cancer cells induces IL-6 secretion and plays an important role in tumorigenesis in an animal model^19^. In the present study, we further demonstrate the effects of MUC2 on migration and metastasis in human colon cancer cells. A previous study indicated that loss of MUC2 expression was observed during the adenoma-carcinoma sequence^8^. The MUC2 promoter contains binding sites for multiple transcription factors, and the contribution of the STAT3/CREB/ATF1/Chk2/TP53 axis to this mechanism is possibly dependent on the molecular signature of cancer cells. A summary of the experimental results is presented in Fig. 6c. The list of relevant interacting proteins retrieved from the STRING database comprises MUC2, IL-6, STAT3, CREB1, CHEK2, CDH1, and TP53^26^. The MUC2 protein plays an important role in IL-6 signaling during colon cancer metastasis. To our knowledge, this is the first study to report that MUC2 directly mediates Chk2/STAT3/CREB/ATF-1 signaling and E-cadherin expression in colon cancer. This study is the first to use pathway and network-assisted analyses of MUC2, which provide a great deal of important information regarding protein interactions and regulation in nonmucinous colon AC. The results suggest potential functional components underlying the molecular mechanisms of MUC2 and may facilitate a better understanding of the signaling networks involved in metastasis.

Both autocrine and paracrine IL-6 signaling mechanisms promote the invasion and metastasis of human tumors^24^. The inflammatory cytokine IL-6 promotes EMT, which contributes to the invasiveness of cancer cells^24^. STAT3 is significantly associated with poor prognosis in stage II colon cancer patients^27^. Oxaliplatin and camptothecin block IL-6 by inhibiting the interaction between STAT3 and gp130^27^. In the present study, IL-6 increased the phosphorylation of STAT3 in MUC2-silenced HT-29 cells. MUC2 participates in STAT3-induced cell migration and E-cadherin downregulation in HT-29 cancer cells. Conversely, decreased MUC2 mRNA correlates significantly with worse survival in patients with hepatocellular carcinoma (HCC)^28^. The MUC2 mRNA expression in HCC cell lines is increased by the epigenetic inhibitors 5-aza-2′-deoxycytidine and Trichostatin A^28^. Thus, our results suggest that MUC2 may serve as a novel therapeutic target for suppressing liver metastases of the colon.

Aberrantly glycosylated in malignancies, mucins mediate cancer cell interactions with leukocytes present in the tumor microenvironment during metastasis^29^. Tumor-associated macrophages are the principal leukocyte subset. Colon cancer cells stimulate macrophages to produce IL-6, which activates STAT3 in tumor cells^30^. STAT3 activation induces IL-6 expression in both tumor and stromal cells and creates a tumor-promoting inflammatory microenvironment^31^. In the present study, we demonstrated that MUC2 suppression induces IL-6-mediated phosphorylation of STAT3. The silencing of MUC2 in HT-29 cells increased gp130 expression. The IL-6-gp130-STAT3 axis was fundamentally required for the pro-inflammatory process. The deregulation of MUC2 production contributes to chronic inflammation and tumorigenesis^32^. IL-6 signaling is mediated via classic signaling and trans-signaling. Classic signaling is restricted to target cells expressing both the membrane-bound IL-6 receptor alpha (mIL-6R, CD126) subunit and gp130 (CD130), which is widely expressed. Trans-signaling occurs when a soluble form of IL-6R (sIL-6R) enables IL-6 signaling in cells that do not express mIL-6R. Both classic signaling and trans-signaling are mediated through gp130-induced STAT3 phosphorylation^24^. STAT3 activation plays an important role in tumor promotion and progression through the induction of various target genes involved in tumor cell survival (e.g., Bcl-2, Survivin, Mcl-1), proliferation (e.g., c-Myc, Cyclin D1, Cyclin B), angiogenesis (e.g., HIF1α, VEGF), metastasis (e.g., MMP2, MMP9), cell adhesion (e.g., ICAM-1, TWIST1), inflammation (e.g., IL-6, IL-17, IL-23, Cox2) and other processes^33, 34^. The proinflammatory activities of IL-6 and tumorigenesis are mediated by IL-6 trans-signaling^35^. IL-6 trans-signaling results in strong phosphorylated STAT3 expression and a significantly increase tumor-associated antigens carcinoembryonic antigen-related cell adhesion molecule 5/6 (CEACAM5/6) expression^36^. CEACAM5/6 are involved in cell adhesion, migration, tumor invasion and metastasis^37, 38^. Our results imply that MUC2 downregulation is associated with increased expression of the tumor-associated antigens CEACAM5/6 in colon cancer.

In conclusion, this study is the first to examine the interaction between MUC2 and IL-6 protein expression in human colon cancer. Our results demonstrate that MUC2 downregulation and IL-6 overexpression correlate with human colon cancer metastasis. These data suggest that elevated expression of MUC2 inhibits EMT and metastasis. Our results indicate that analyzing MUC2 expression in stage II colon cancer patients might help identify those who would potentially benefit from targeted therapy. Thus, MUC2 expression is a valuable prognostic indicator for colon cancer.

## Materials and Methods

### Patients

Formalin-fixed, paraffin-embedded sections of tissue samples from 102 patients with stage IIA colon cancer were collected and stored in the Human Biobank within the Research Center of Clinical Medicine of the National Cheng Kung University Hospital (NCKUH). These patients were randomly selected between August 2005 and August 2010. The demographics, histopathologic findings, and clinical outcomes were collected through a retrospective review of each patient’s medical chart. The pathologic staging was determined in accordance with the tumor, node, metastasis (TNM) staging system of the American Joint Committee on Cancer (AJCC) staging manual^39^. All patients provided written informed consent, and the study was approved by the Institutional Review Board of NCKUH (NCKUH IRB number: ER-97-148). All experiments were performed in accordance with the guidelines by the Institutional Review Board.

### Immunohistochemical analysis of MUC2 and IL-6 expression

Formalin-fixed, paraffin-embedded sections of colon cancer lesions were analyzed using immunohistochemistry as previously described^40^. Sections (4 μm) were cut from each tissue array block. The sections were deparaffinized and dehydrated. After the sections were subjected to antigen retrieval in an autoclave, immunohistochemical staining was performed by incubating the sections overnight with a mouse anti-human MUC2 antibody (Ccp58; 1:100 dilution; Novocastra, Newcastle upon Tyne, England), a mouse anti-human IL-6 antibody (3G9; 1:100 dilution; Origene Technologies, Inc., Rockville, MD) and a mouse anti-human IL-68 antibody (clone KP-1; 1:100 dilution; code No. M0814; DAKO). The sections were then incubated with an avidin-biotin complex reagent (DAKO, Carpinteria, CA, USA), and the final color was developed with 3-amino-9-ethyl carbazole (AEC) (Zymed Laboratories, Inc., San Francisco, CA, USA). The sections were counterstained with hematoxylin. The immunoreactivity of the MUC2, IL-6 and CD68 proteins was assessed using a semiquantitative method and scaled according to the immunoreactive score (IRS) of Remmele and Schicketanz^41^. The sections were divided into four categories on the basis of the IRS scores, which ranged from 0 to 12: negative, weak, mild, and strong. Dr. H.P. Hsu assessed the lesions.

### Cell culture

Human HT-29, LS174T, SW620, LoVo, and Colo205 colon AC cells were kindly provided by the Clinical Medicine Research Center and Dr. MD Lai (National Cheng Kung University, Tainan, Taiwan). LS174T cells were maintained in MEM medium containing 10% fetal bovine serum (FBS) and 1% penicillin/streptomycin. HT-29 cells were maintained in McCoy’s 5a medium containing 10% FBS and 1% penicillin/streptomycin. SW620, LoVo and Colo205 cells were maintained in Dulbecco’s Modified Eagle Medium (DMEM)/high glucose containing 10% FBS (HyClone Laboratories, Logan, UT) and 1% penicillin/streptomycin.

### RNA interference, transfection, and stable cell line generation

For shRNA-mediated signaling, shRNAs targeting MUC2 and STAT3 were purchased from the National RNAi Core Facility (Academia Sinica, Taiwan; http://rnai.genmed.sinica.edu.tw) and inserted into the pLKO.1 plasmid. A control construct (pLKO.1 containing a luciferase non-silencing shRNA) was also purchased. The following target sequences were used: human MUC2, 5′-GCTCTCCAATAACCACCACAA-3′ (RNAi-1) and 5′-CGACTACAAGATACGTGTCAA-3′ (RNAi-2); human STAT3, 5′-CCTGAGTTGAATTATCAGCTT-3′ (RNAi-1) and 5′-GCAGGTATCTTGAGAAGCCAA-3′ (RNAi-2). HT-29 and LS174T cells were transfected with the luciferase non-silencing shRNA and vectors containing shRNAs corresponding to the target sequences using the transfection reagent Lipofectamine 2000 (Invitrogen, Life Technologies, Darmstadt, Germany) as previously described^42^. Single cell clones of the transfectants were selected using the limiting dilution method. We used two different MUC2 shRNAs to establish stable MUC2 knockdown clones of HT-29 and LS174T cells via puromycin (1 µg/mL) (Sigma) selection. Two individual clones were obtained from HT-29 and LS174T cells for each shRNA and were termed shMUC2-1.1, shMUC2 -1.2, shMUC2 -2.1, and shMUC2 -2.2. We also used two different STAT3 shRNAs to establish stable STAT3 knockdown clones of HT-29 cells via puromycin (1 µg/mL) selection. Two individual clones were obtained from HT-29 cells for each shRNA and were termed shSTAT3-1 and shSTAT3-2. The selected stable clones were maintained in complete medium containing puromycin. For the stable transfectants, three seedings were performed. To monitor the efficacy of MUC2 silencing, MUC2 expression in the stable transfectants was analyzed by western blotting. For gp130 (CD130) detection, 1 × 10^6^ tumor cells were incubated for 10 min at 4 °C with 1 μg/μL PE-conjugated mouse antihuman CD130 monoclonal antibody or PE-conjugated mouse IgG1 isotype control. Stained tumor cells were analyzed by flow cytometry (FACScan; BD Biosciences, San Jose, CA).

### Western blot analysis

Total cell lysates were prepared and analyzed by SDS-PAGE as previously described^43^. For quantification, the bands were measured using an AlphaImager 2200 system (Alpha Innotech, San Leandro, CA, USA) and were normalized to the band density of β-actin. MUC2 expression was quantified and is presented as the MUC2 to β-actin ratio. These experiments were repeated using three independent batches of cell clones or cell lysates. The quantitative data are presented as the values relative to those in control cells. For more detailed information, please see the Supporting Information.

### Cell proliferation assay

HT-29 (5 × 10^3^ cells/well) and LS174T (5 × 10^3^ cells/well) cells were seeded in triplicate in 96-well plates and incubated at 37 °C in 5% CO_2_. At daily intervals (24, 48, and 72 h), the number of viable cells was measured using the CellTiter 96 Aqueous One Solution cell proliferation assay (Promega, Madison, WI, USA) according to the manufacturer’s instructions. Then, 20 μL of CellTiter One Solution was added to each well. After a 1 h incubation, the absorbance at 490 nm was recorded using an ELISA plate reader.

### Collection of macrophage supernatants and tumor cell culture conditions

PBMCs were obtained from the buffy coat of whole blood from healthy donors. The PBMCs were separated on Ficoll–Paque (Amersham Biosciences, Piscataway, NJ, USA) and centrifuged at 2400 rpm for 20 min at room temperature (RT). The culture supernatants were collected. For more detailed information, please see the Supporting Information.

### Human phospho-kinase array assay

The level of phosphorylation was determined in cell lysates collected from one million vector control and shMUC2-2.2 cells that were treated with recombinant human IL-6 (25 ng/mL) (R&D Systems, Minneapolis, MN, USA) for 30 min. Relative phosphorylation levels were determined using Human Phospho-RTK Arrays (R&D Systems, Minneapolis, MN, USA) according to the manufacturer’s instructions. Cell lysates were used immediately or stored at −80 °C and were incubated with a human phosphorylation array panel antibody cocktail at RT for 1 h. After blocking, the membranes were incubated with cell lysates at 4 °C overnight on a rocking platform. The membranes were subsequently washed and incubated with a freshly diluted detection antibody cocktail for 2 h at RT on a rocking platform. The membranes were subsequently washed and incubated with diluted streptavidin conjugated to horseradish peroxidase (HRP) for 30 min at RT on a rocking platform. The membranes were developed with Chemi Reagent Mix and exposed to X-ray film. The signal intensity of the array was scanned and quantified by densitometry using an AlphaImager 2200 system (Alpha Innotech) and normalized to the positive control.

### Quantification of IL-6, phospho-STAT3 (Y705), phospho-CREB (S133), and phospho-Chk2 (T68) by ELISA

IL-6 levels were measured in MSs and tumor cell culture supernatants using commercial ELISA kits (R&D Systems, Abingdon, U.K.), according to the manufacturer’s instructions. For more detailed information, please see the Supporting Information.

### Wound-healing assay

To evaluate the motility of the HT-29 and LS174T cell clones, *in vitro* wound-healing assays were performed^42^. HT-29 cells (70 μL of 1 × 10^6^ cells/mL) were seeded in an ibidi culture insert (Applied BioPhysics, Inc., Martinsried, Germany) above a 6-well plate. After incubating overnight, the cell culture insert was carefully removed to form a cell-free gap between the attached cells. The incubation time for the wound-healing assay depended on the tumor cells used. Cell motility into this defined wound was observed and analyzed. Six fields were randomly selected, and the number of migrated cells was counted.

### Mice and ethics statement

Six-to eight-week-old BALB/c and NOD/SCID mice were purchased from the Laboratory Animal Center of National Cheng Kung University (Tainan, Taiwan) and were maintained under specific pathogen-free conditions. The animal experiments were approved by the Institutional Animal Care and Use Committee of National Cheng Kung University. The methods were performed in accordance with the approved guidelines (Approval No: CN-IACUC-102013).

### Experimental metastatic model of colon carcinoma

The metastatic abilities of the HT-29 and CT26 cell clones *in vivo* were evaluated using a hepatic metastasis model, in which 1 × 10^6^ tumor cells in 0.05 mL of PBS were injected intrasplenically as previously described^42, 43^. For more detailed information, please see the Supporting Information.

### Statistical analysis

In the present study, we conducted a search of the Oncomine database (http://www.oncomine.com) to systematically assess the expression levels of the *MUC2* and *IL-6* genes in colonic cancer^44^. We compiled information on the expression of *MUC2* and *IL6* in normal and cancerous colonic tissues from all the microarray studies in the database^45–51^. The relationship between *MUC2* and *IL-6* gene expression and prognosis in patients with colon cancer was examined using the PrognoScan database^52^. The patient samples were split into high and low expression groups with the optimal cutoff point determined according to the database description. In the graphs presented in the figures, the x-axis represents time, and the y-axis represents survival rate. The P-value was calculated using Pearson’s linear correlation. The protein interactions were downloaded from STRING version 8.0 on December 19, 2008 (http://string.embl.de). All statistical analyses were performed using SPSS version 12.0 (SPSS Institute, Chicago, IL, USA). Univariate analysis between categorical variables was performed using the Chi-square test. Continuous variables that did not follow the normal distribution were compared using the nonparametric Mann–Whitney or Kruskal–Wallis test. P < 0.05 was considered statistically significant. The data are presented as the mean ± the standard deviation (SD). Statistical analyses between two groups were performed using Student’s *t*-test. One-way ANOVA was used for multiple group comparisons.

## Electronic supplementary material

Supplementary Information
