# Supplementary material for: Mucin 2 silencing promotes colon cancer metastasis through interleukin-6 signaling
Source: Sci Rep. 2017 Jul 19;7:5823. doi: 10.1038/s41598-017-04952-7 (PMC5517441; doi:10.1038/s41598-017-04952-7)
Supplement: Supplementary file 1 — Supplementary Information [file 41598_2017_4952_MOESM1_ESM.pdf]

Mucin 2 silencing promotes colon cancer metastasis through interleukin-6 signaling

Hui-Ping Hsu[1], Ming-Derg Lai[2,3], Jenq-Chang Lee[1], Meng-Chi Yen[2,4], Tzu-Yang Weng[2,3], Wei-Ching Chen[2,3], Jung-Hua Fang[5], Yi-Ling Chen[6,7*]

[1]Department of Surgery, National Cheng Kung University Hospital, College of Medicine, National Cheng Kung University, Tainan, Taiwan

[2]Department of Biochemistry and Molecular Biology, College of Medicine, National Cheng Kung University, Tainan, Taiwan

[3]Institute of Basic Medical Sciences, College of Medicine, National Cheng Kung University, Tainan, Taiwan

[4]Department of Emergency Medicine, Kaohsiung Medical University Hospital, Kaohsiung Medical University, Kaohsiung, Taiwan

[5]Laboratory Animal Center, College of Medicine, National Cheng Kung University, Tainan, Taiwan

[6]Department of Senior Citizen Service Management, Chia Nan University of Pharmacy and Science, Tainan, Taiwan

[7]Senior Citizen Development Center, Chia Nan University of Pharmacy and Science, Tainan, Taiwan

***Corresponding Author:** Yi-Ling Chen, 60 Erh-Jen Road, Sec 1, Jen-Te, Tainan 71710, Taiwan (ROC), Tel: 886-6-2664911, ext 3716, Fax: 886-6-2664931, Email: s5887110@nckualumni.org.tw

Materials and methods

Antibodies

The following antibodies were used in this study: rabbit anti-MUC2 (EPR6145) (Abcam, Cambridge, MA); mouse anti-E-cadherin (BD Transduction Laboratories, San Jose, CA, USA); rabbit anti-vimentin (Epitomics, Inc); rabbit anti-Chk2, rabbit anti-phospho-Chk2 (Thr 68), rabbit anti-CREB, rabbit anti-phospho-CREB (Ser133), rabbit anti-STAT3, rabbit anti-phospho-STAT3 (Tyr705) and goat anti-rabbit IgG peroxidase conjugate (Cell Signaling, Boston, MA, USA); mouse anti-β-actin (GeneTex, Inc., San Antonio, TX); and sheep anti-mouse IgG peroxidase conjugate (Chemica, San Diego, CA, USA).

Western blot analysis

Total cell lysates were prepared and analyzed by SDS-PAGE as previously described[1]. Tumor cells were washed twice with PBS and lysed with ice-cold RIPA buffer (20 mM Tris-HCl, pH 7.5, 1 mM EDTA, 150 mM NaCl, 1% NP-40 and 1% SDS) containing one protease inhibitor tablet (cOmplete, Mini, EDTA-free Protease Inhibitor Cocktail, Roche; Mannheim, Germany). The tumor cells were harvested using a cell scraper and collected in an eppendorf tube for 20 min at 4°C. The sample was centrifuged for 10 min at 13000 rpm at 4°C, and the supernatant was transferred to an eppendorf tube. The total protein concentrations in the cell lysates were quantitatively assessed using the Bio-Rad Bradford assay (Mississauga, ON).

Immunodetection was performed using an HRP-based SuperSignal Chemiluminescent Substrate (Pierce, Rockford, IL, USA). For quantification, the bands were measured using an AlphaImager 2200 system (Alpha Innotech, San Leandro, CA, USA) and were normalized to the band density of β-actin. MUC2 expression was quantified and is presented as the MUC2 to β-actin ratio. These experiments were repeated using three independent batches of cell clones or cell lysates. The quantitative data are presented as the values relative to those in control cells.

Collection of macrophage supernatant and tumor cell culture conditions

Peripheral blood mononuclear cells (PBMCs) were obtained from the buffy coat of whole blood from healthy donors. The PBMCs were separated on Ficoll–Paque (Amersham Biosciences, Piscataway, NJ, USA) and centrifuged at 2400 rpm for 20 min at room temperature (RT). The culture supernatants were collected. The PBMCs were resuspended in RPMI 1640 medium supplemented with 10% FBS. Macrophage-like cells were allowed to adhere to culture plates at 37°C in an incubator in a humidified atmosphere of 5% CO_2 for 2 h. Nonadherent cells were removed by washing the plate with RPMI 1640 medium, and adherent cells were harvested and plated at 4×10^5 cells each well for 48 hours. Adherent cells were cultured in wells containing 2 mL RPMI 1640 with 10% FBS. To confirm the purity of macrophage-like cells, adherent cells were collected and incubated with anti-CD14-FITC at

4°C for 30 min in the dark. Stained adherent cells were analyzed by flow cytometry (FACScan; BD Biosciences, San Jose, CA). The macrophage supernatant (MS) was collected by centrifugation at 1500 rpm for 5 minutes and stored as aliquots at −80°C until use. To compare the IL6 secretions of HT-29–derived cell clones, 4×10^5 HT-29–derived cell clones cultured in six-well plates containing 1 mL McCoy's 5A modified medium per well were incubated with an equal volume of MS for 48 hours. The MS was cultured alone as a control. The culture supernatants were collected, and IL-6 concentrations were detected by ELISA.

IL-6 neutralization assay

To block the effects of IL-6, macrophage supernatants were pretreated with 5 μg/mL of anti-IL-6 antibody (clone 6708, R&D Systems) for 30 minutes before addition to HT-29-derived cells as described[2].

Quantification of IL-6, phospho-STAT3 (Y705), phospho-CREB (S133), and phospho-Chk2 (T68) by ELISA

IL6 levels were measured in MS and tumor cell culture supernatant by ELISA using commercial kits (R&D Systems, Abingdon, U.K.) according to the manufacturer's instructions. No IL-6 was detected in supernatant from the HT-29 cells and HT-29–derived cell clones. To analyze fold change/control, IL-6 levels in the supernatants of HT-29–derived cell clones

cultured with MS were compared to those of HT-29–derived cell clones cultured with MS alone for each of three independent experiments. Subsequently, HT-29–derived cell clones were stimulated with recombinant human IL-6 (25 ng/mL) for 30 min, tested with the cell-based human/mouse phospho-STAT3 (Y705) (R&D Systems), human/mouse/rat phospho-CREB (S133) (R&D Systems), and human phospho-Chk2 (T68) (R&D Systems) ELISA kit, and analyzed by western blot.

Experimental metastatic model of colonic carcinoma

CT26 colon carcinoma cells (BALB/c mouse origin) were maintained in high glucose Dulbecco's modified Eagle's medium (DMEM) (Gibco-Invitrogen) containing 10% FBS (HyClone Laboratories, Logan, UT, USA) and 1% penicillin/streptomycin. The metastatic abilities of HT-29 and CT26 cell clones *in vivo* were evaluated using a hepatic metastasis model in which 1×10^6 tumor cells in 0.05 mL of PBS were intrasplenically injected as previously described[1,3]. Fourteen days after HT-29 cell clone injection, the NOD/SCID mice were sacrificed, and liver metastases were examined macroscopically and histologically. Fourteen days after CT26 cell clone injection, the BALB/c mice were sacrificed, and liver metastases were examined macroscopically and histologically. Formalin-fixed, paraffin-embedded sections of the liver and spleen were used for hematoxylin and eosin (H&E) staining. Each animal experiment was performed at least twice.

References

1 Shan, Y. S. *et al.* Argininosuccinate synthetase 1 suppression and arginine restriction inhibit cell migration in gastric cancer cell lines. *Sci Rep* **5**, 9783 (2015).

2 Li, Y. Y., Hsieh, L. L., Tang, R. P., Liao, S. K. & Yeh, K. Y. Macrophage-derived interleukin-6 up-regulates MUC1, but down-regulates MUC2 expression in the human colon cancer HT-29 cell line. *Cell Immunol* **256**, 19-26 (2009).

3 Shan, Y. S. *et al.* Suppression of mucin 2 promotes interleukin-6 secretion and tumor growth in an orthotopic immune-competent colon cancer animal model. *Oncol Rep* **32**, 2335-2342 (2014).

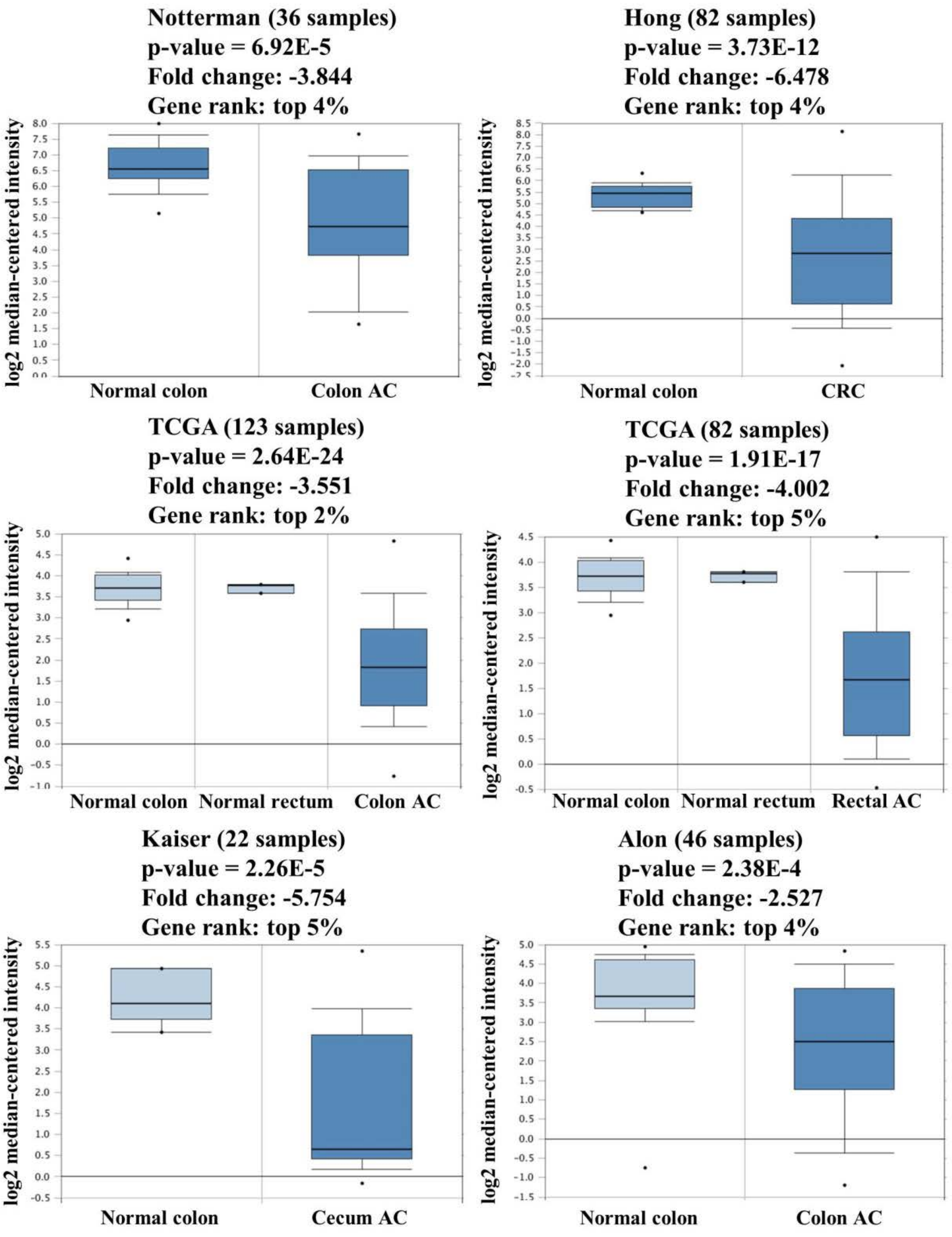

Supplementary Figure S1-1. ***The mucin-2 (MUC2)*** **gene was underexpressed in colonic cancer.** The expression patterns of *MUC2* in colonic cancer datasets were obtained from the Oncomine database. AC, adenocarcinoma; CRC, colorectal carcinoma.

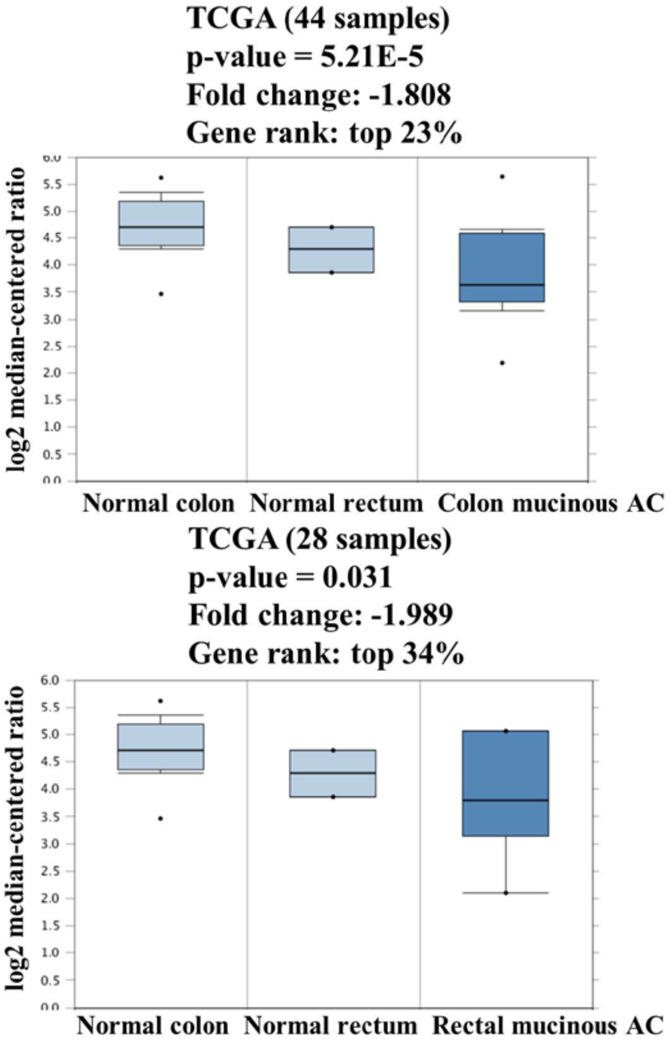

Supplementary Figure S1-2. Continued.

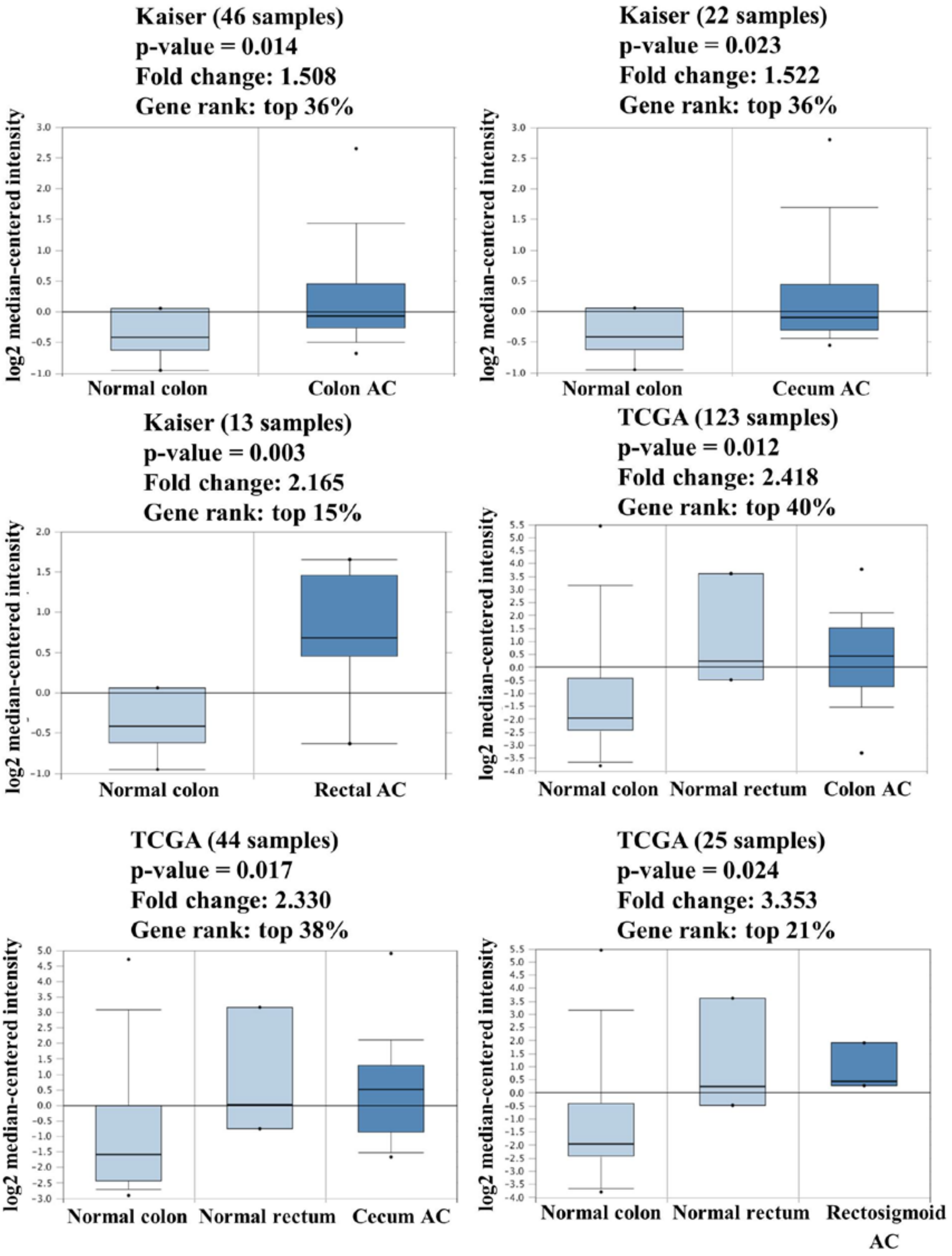

Supplementary Figure S2-1. ***The interleukin-6 (IL-6)*** **gene was overexpressed in colonic cancer.** The expression patterns of *IL-6* in colonic cancer datasets were obtained from the Oncomine database. AC, adenocarcinoma; CRC, colorectal carcinoma.

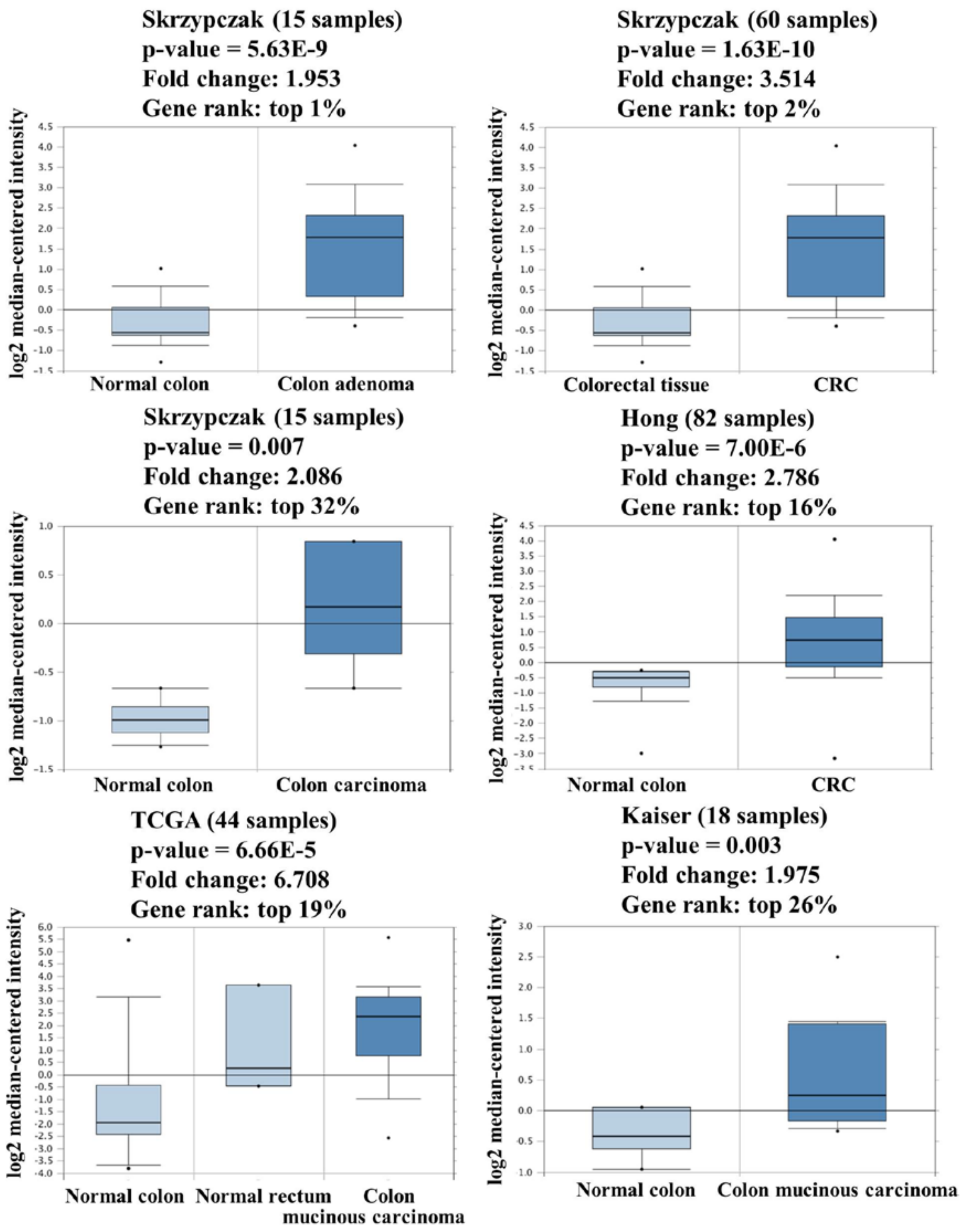

Supplementary Figure S2-2. Continued.

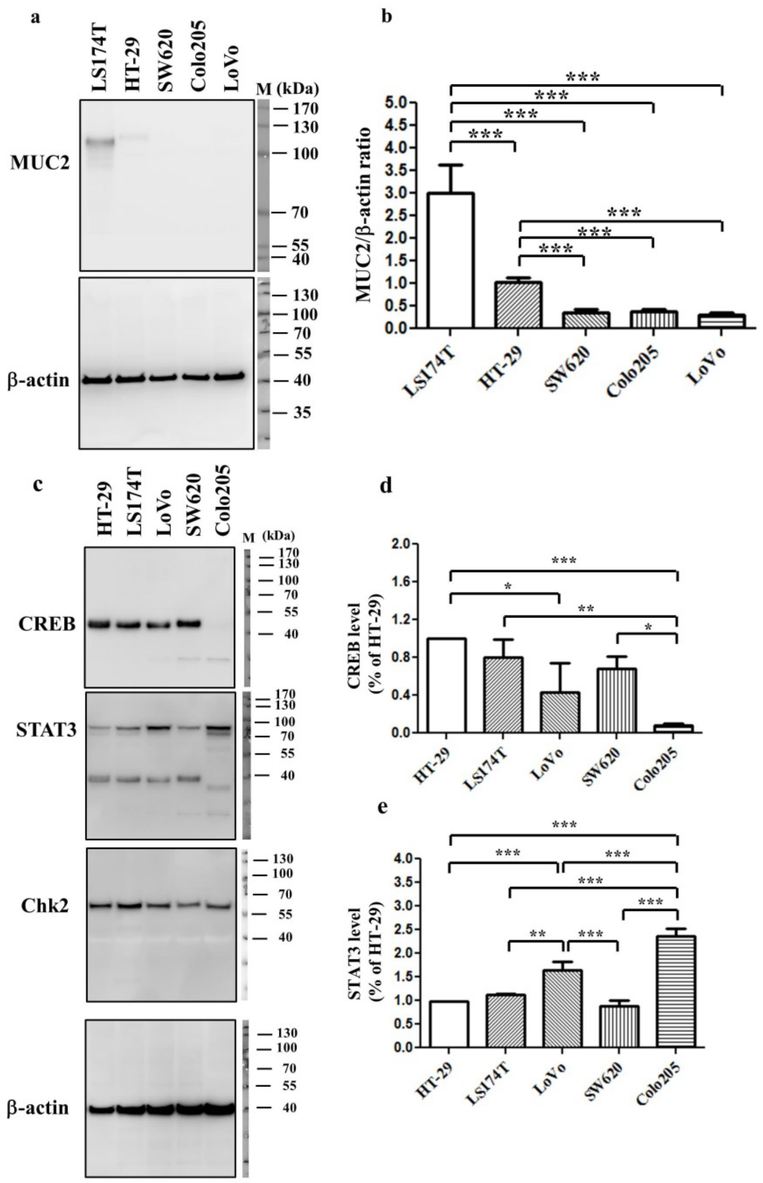

Supplementary Figure S3. The protein expression levels of MUC2, CREB, STAT3 and Chk2 in colon cancer cell lines. (a&b) MUC2 protein expression was determined in LS174T, HT-29, SW620, Colo205, and LoVo cells. (c) The protein expression levels of CREB, STAT3, and Chk2 were determined in HT-29, LS174T, LoVo, SW620, and Colo205 cells. (d) CREB level (e) STAT3 level. The bars represent the mean ± SD. The results were obtained from three independent experiments. $^{**}P < 0.001$; $^{***}P < 0.0001$.

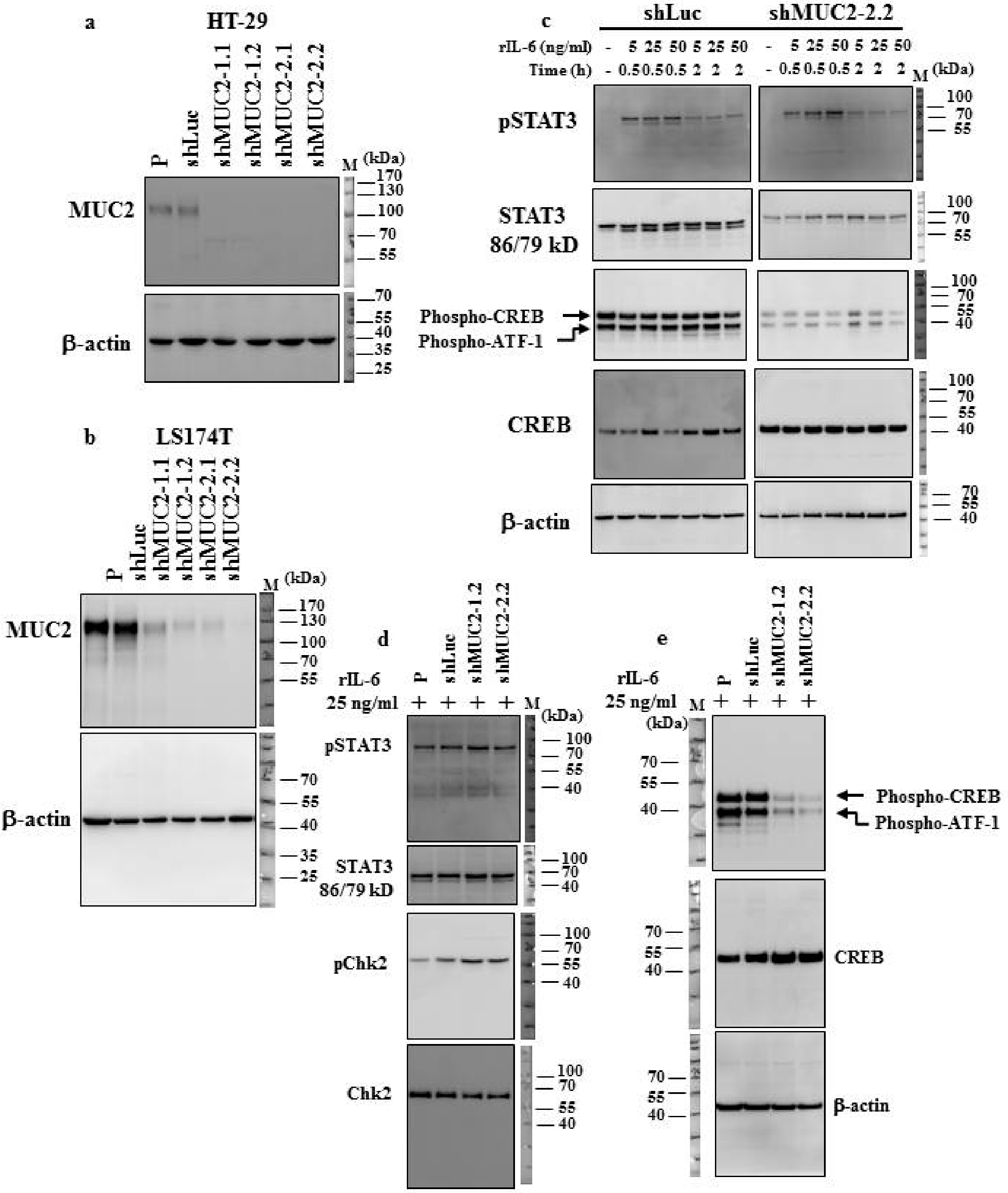

Supplementary Figure S4. Cell lysates were immunoblotted using anti-MUC2, anti-pSTAT3, anti-STAT3, anti-pCREB, anti-CREB, anti-pChk2, anti-Chk2, and anti-β-actin antibodies. These cropped blots are shown in Fig. 2a, Fig. 2b, Fig. 5a and Fig. 5b.

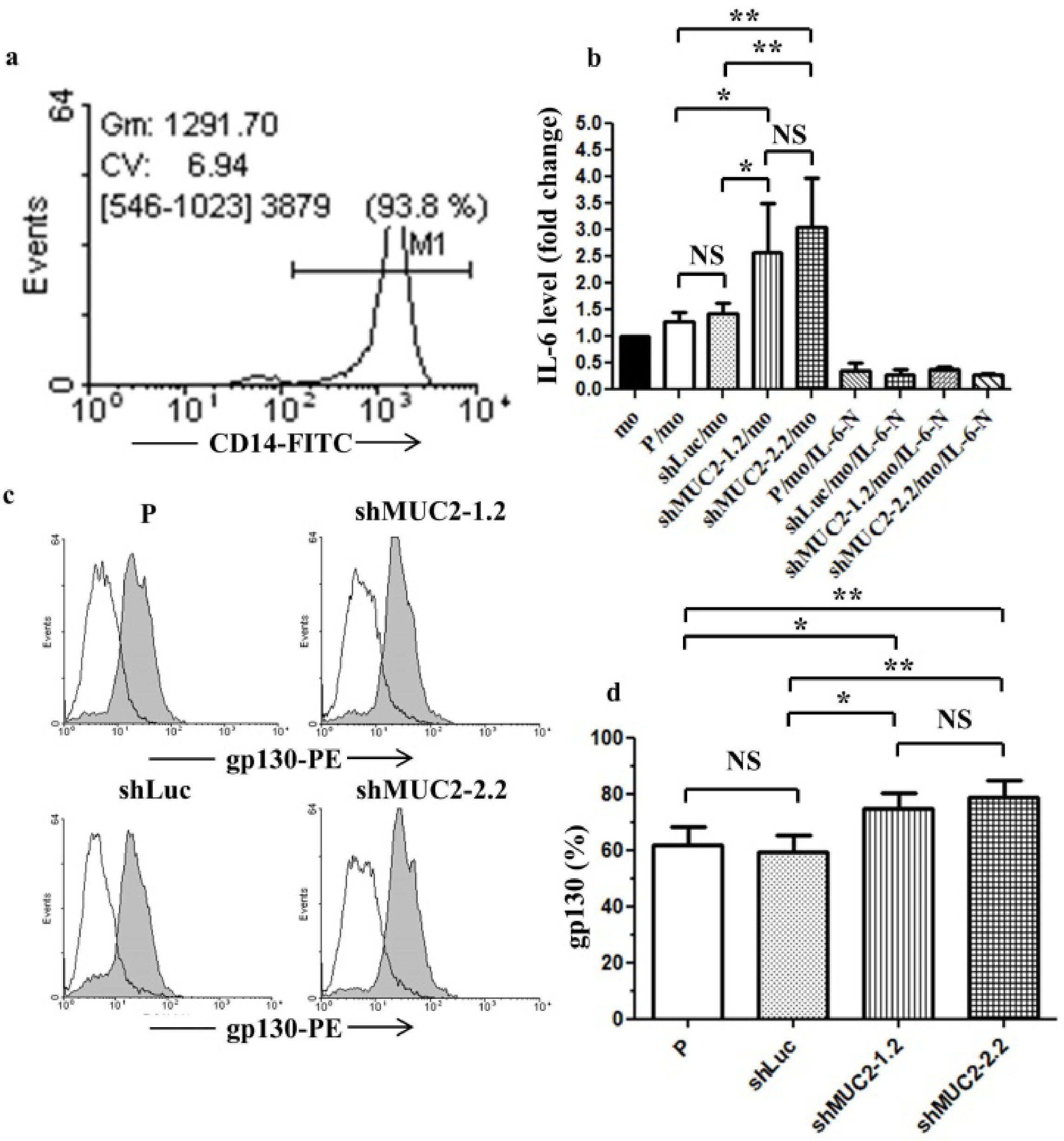

Supplementary Figure S5. MUC2 silencing HT-29 cells increases IL-6 secretion after macrophage supernatant treatment. (a) $CD14^+$ cells were evaluated in the adherent cells from peripheral blood mononuclear cells. (b) Silencing of MUC2 in HT-29 cells enhances IL-6 secretion after treatment with macrophage supernatant (MS). IL-6 protein levels in the MS and HT-29-derived cell clones cultured with MS for 48 hours were measured by ELISA. MS was treated with 5 μg/ml anti-IL-6 antibody (IL-6-neutralization) for 30 minutes before being

added to the HT-29-derived cells. (c) HT-29-derived cells were harvested and stained with a PE-conjugated antihuman gp130 mAb and examined by flow cytometry. Histograms represent the cell surface expression of gp130 (shaded). A PE-conjugated isotype control mAb (unshaded) was used as a negative control. (d) $gp130^+$ cells were evaluated in the HT-29–derived cell clones. NS, not significant; $^{*}P < 0.01$; $^{**}P < 0.001$; $^{***}P < 0.0001$.

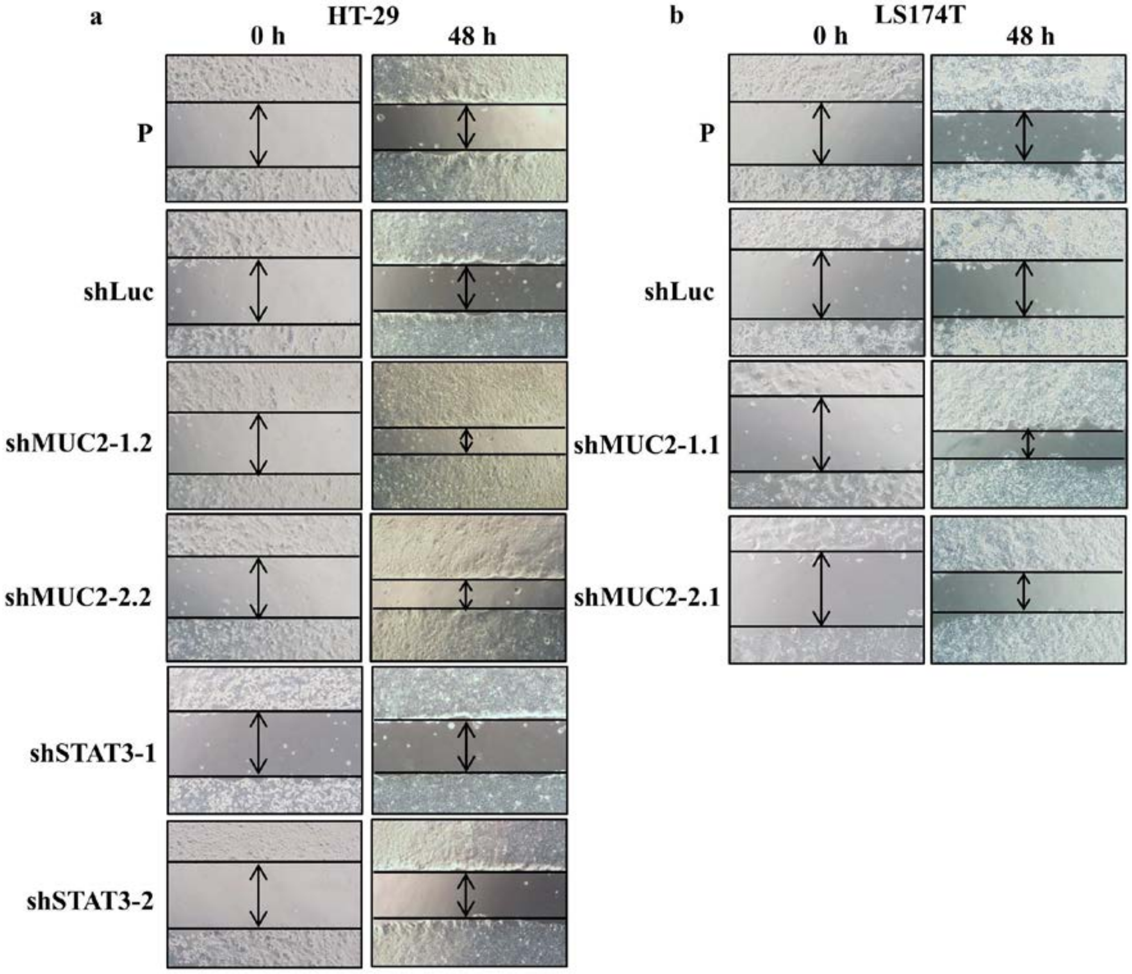

Supplementary Figure S6. The level of MUC2 expression in colon cancer cell lines affected cell migration. MUC2 silencing in HT-29 (a) and LS174T (b) cell clones increased cell migration *in vitro*. Representative micrographs of one of the individual experiments at time points 0 and 48 h are shown. Cell motility toward the gap area was photographed after 48 h. P, parental cells; shLUC, luciferase non-silencing shRNA; shMUC2-1.2 and shMUC2-2.2, MUC2-specific shRNAs 1 and 2, respectively; shSTAT3-1 and shSTAT3-2, STAT3-specific shRNAs 1 and 2, respectively.

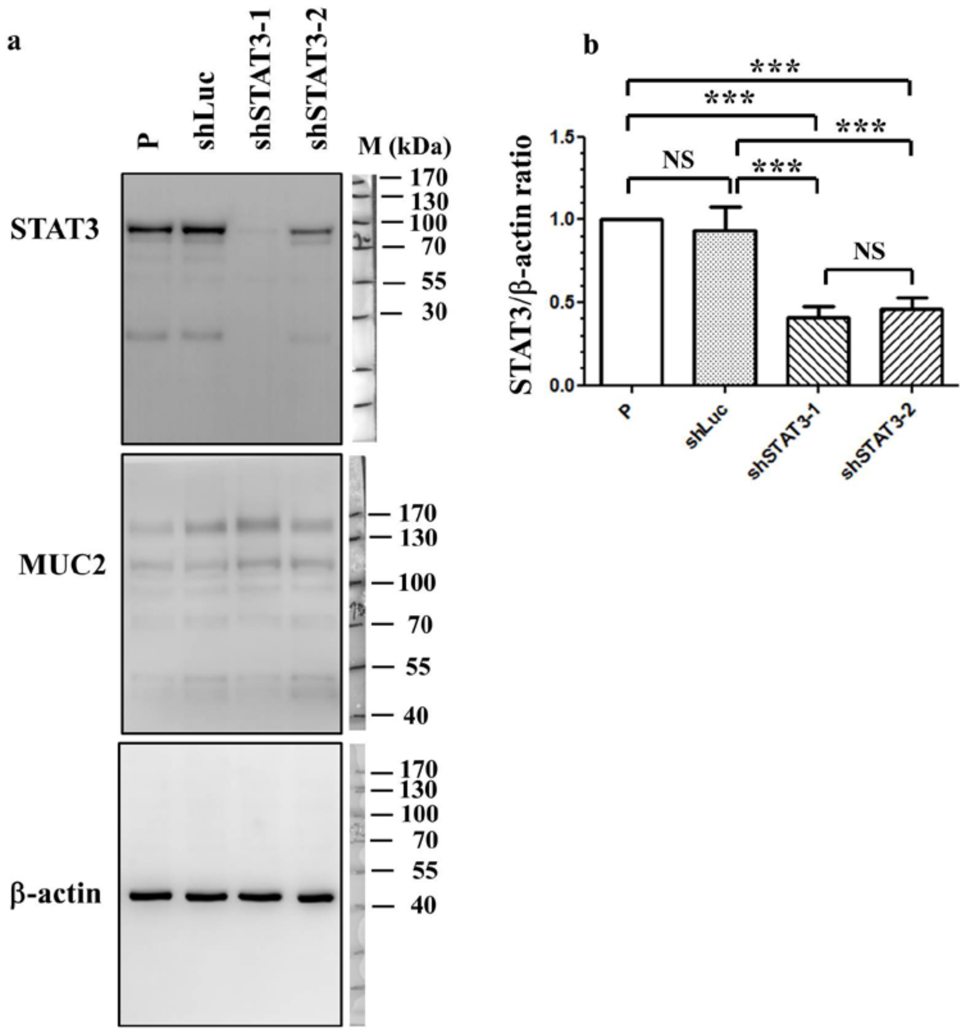

Supplementary Figure S7. STAT3 silencing in HT-29 cell clones decreased STAT3 protein expression. (a&b) STAT3 and MUC2 protein expression was determined in human HT-29 cells and STAT3 shRNA stable transfectants. The results of western blot analysis of protein expression obtained from three independent experiments. Bars represent the mean ± SD. P, parental cells; shLuc, luciferase non-silencing shRNA; shSTAT3-1 and shSTAT3-2, STAT3-specific shRNAs 1 and 2, respectively; shMUC2-1.2 and shMUC2-2.2, MUC2-specific shRNAs 1 and 2, respectively. $^{*}P < 0.01$; $^{**}P < 0.001$; $^{***}P < 0.0001$.

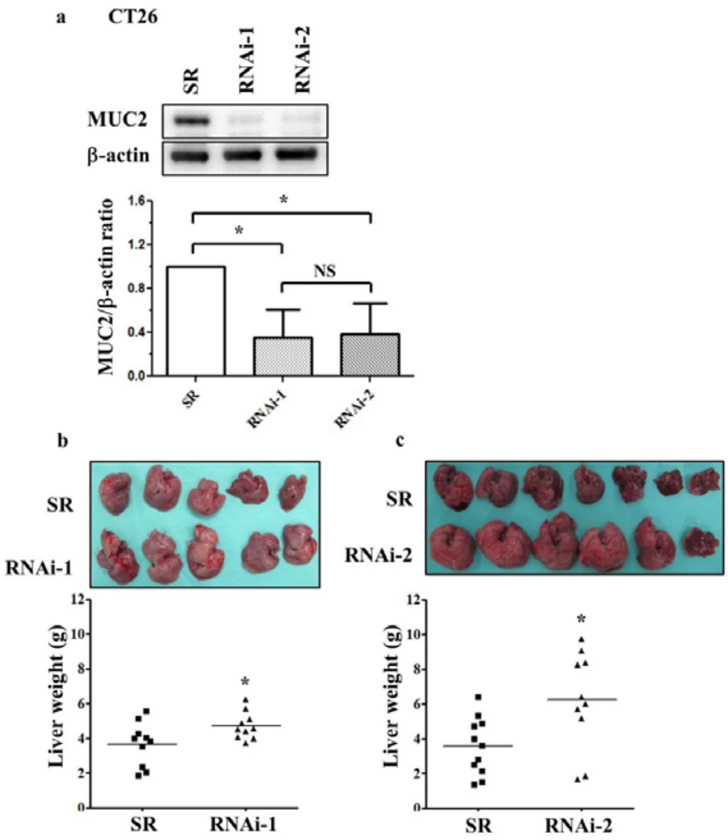

Supplementary Figure S8. MUC2 silencing in CT26 cell clones suppressed tumor metastatic ability *in vivo*. (a) MUC2 protein was determined in scramble RNA (SR), MUC2 RNAi-1 and MUC2 RNAi-2 CT26 cell clones. The results of western blot analysis of protein expression were obtained from three independent experiments. The bars represent the mean ± SD. (b) MUC2 silencing in CT26 cell clones enhances metastasis *in vivo*. (b&c) Tumor masses from the liver were macroscopically visualized after intrasplenic injection of SR, MUC2 RNAi-1 and MUC2 RNAi-2 CT26 cell clones. Weights of the liver of BALB/c mice injected with colon cancer cells on day 14. Results are expressed as the mean ± SD of two independent experiments. $^{*}P < 0.05$.

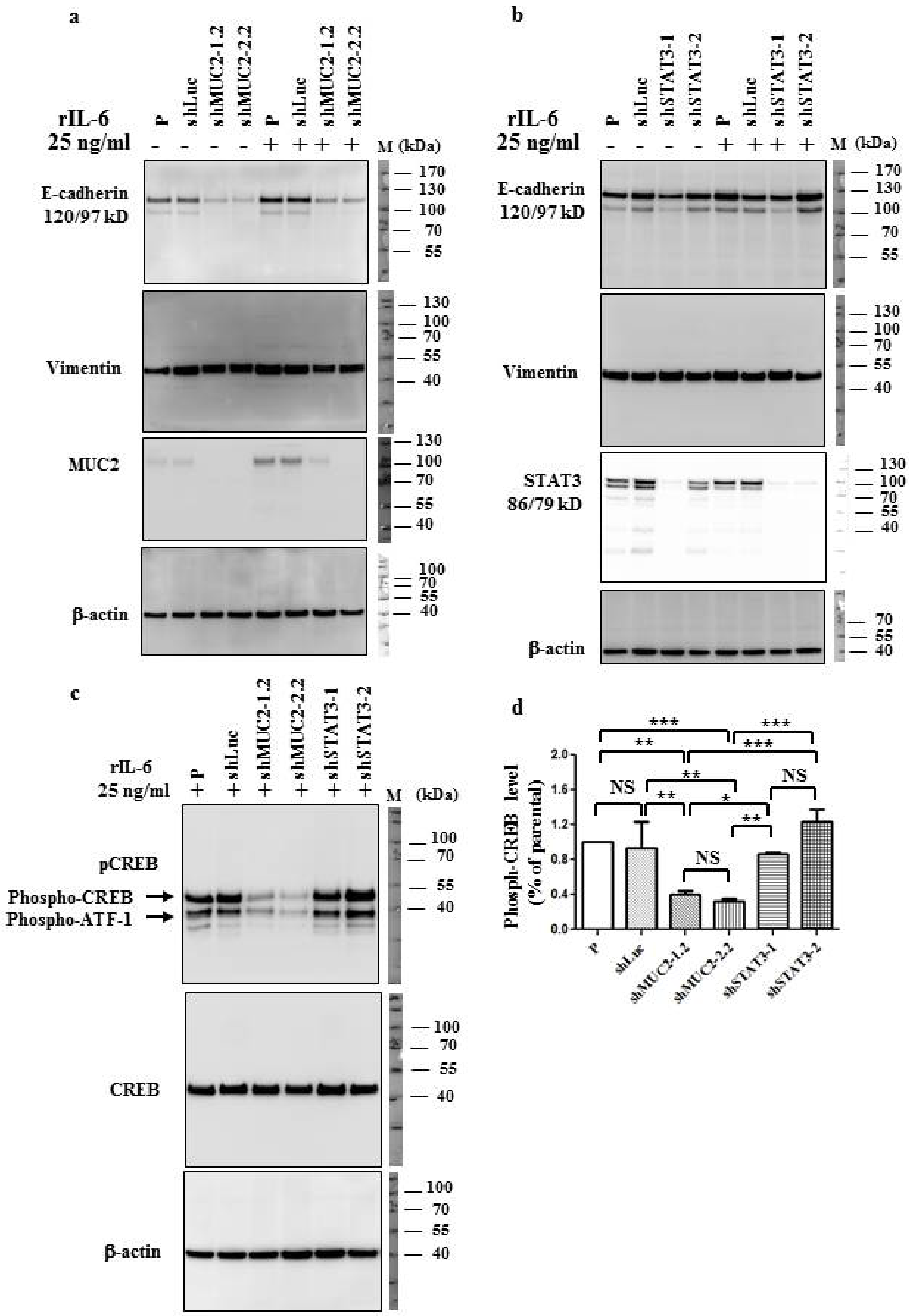

Supplementary Figure S9. Cell lysates were immunoblotted using anti-E-cadherin, anti-vimentin, anti-MUC2, anti-STAT3 and anti-β-actin antibodies. These cropped blots are shown in Fig. 6a and Fig. 6b.

Supplementary Table 1. Demographics and histopathological findings in patients with stage II colon cancer

	**Expression of MUC2**			**Expression of IL-6**		
	**Negative**	**Positive**	**P-value**	**Negative**	**Positive**	**P-value**
**Patient, n (%)**	14 (14%)	88 (86%)		53 (52%)	49 (48%)	
**Gender, n (%)**			>0.999			0.067
**Female**	5 (13%)	34 (87%)		25 (64%)	14 (36%)	
**Male**	9 (14%)	54 (86%)		28 (44%)	35 (56%)	
**Histological differentiation**			0.149			0.698
**Mucinous carcinomas**	0	12 (100%)		7 (58%)	5 (42%)	
**Well-differentiated adenocarcinoma**	3 (23%)	10 (77%)		5 (39%)	8 (61%)	
**Moderately-differentiated adenocarcinoma**	11 (15%)	63 (85%)		39 (53%)	35 (47%)	
**Poorly-differentiated adenocarcinoma**	0	3 (100%)		2 (67%)	1 (33%)	
**Recurrence pattern**			0.182			0.411
**Liver metastasis**	0	8 (100%)		3 (38%)	5 (62%)	
**Other metastasis**	2 (29%)	5 (71%)		5 (71%)	2 (29%)	
